# Discovery of amantadine formate: Toward achieving ultrahigh pyroelectric performances in organics

**DOI:** 10.1016/j.xinn.2021.100204

**Published:** 2022-01-01

**Authors:** Junyan Zhou, Shifeng Jin, Congcong Chai, Munan Hao, Xin Zhong, Tianping Ying, Jiangang Guo, Xiaolong Chen

**Affiliations:** 1 Beijing National Laboratory for Condensed Matter Physics, Institute of Physics, Chinese Academy of Sciences, Beijing 100190, China; 2 School of Physical Sciences, University of Chinese Academy of Sciences, Beijing 101408, China; 3 College of Materials Science and Opto-Electronic Technology, University of Chinese Academy of Sciences, Beijing 101408, China; 4 Materials Research Center for Element Strategy, Tokyo Institute of Technology, Yokohama 226-8503, Japan; 5 Songshan Lake Materials Laboratory, Dongguan 523808, China

**Keywords:** organic pyroelectric, molecular ferroelectric, phase transition, flexible electronics, negative piezoelectric

## Abstract

Pyroelectrics are a class of polar compounds that output electrical signals upon changes in temperature. With the rapid development of flexible electronics, organic pyroelectrics are highly desired. However, most organics suffer from low pyroelectric coefficients or low working temperatures. To date, the realization of superior pyroelectric performance in all-organics has remained a challenge. Here, we report the discovery of amantadine formate, an all-organic pyroelectric with ultrahigh voltage figures of merit (*F*_v_), surpassing those of all other known organics and commercial triglycine sulfate, LiTaO_3_ as well around room temperature. The key to the high *F*_v_ is attributed to large pyroelectric coefficients in a favorable temperature range resulting from a ferroelectric-paraelectric phase transition of second order at 327 K, small dielectric constant, and moderate heat capacity. In addition, amantadine formate is relatively lightweight, soft, transparent, low-cost, and non-toxic, adding value to its potential applications in flexible electronics. Our results demonstrate that a new type of pyroelectrics can exist in organic compounds.

## Public summary

• Organic pyroelectrics have great potential in wearable devices for temperature sensing, IR detection, thermal imaging, and energy harvesting
• We report the first all-organic pyroelectric amantadine formate with properties better than that of TGS, a hybrid pyroelectric in use since the 1950s
• Amantadine formate has a large pyroelectric coefficient and a surprisingly small dielectric constant, which play a key role in its excellent pyroelectric performance
• The strategy of combining all-organic components and second-order phase transition will contribute to the exploration of new pyroelectrics

## Graphical abstract

**Figure:**
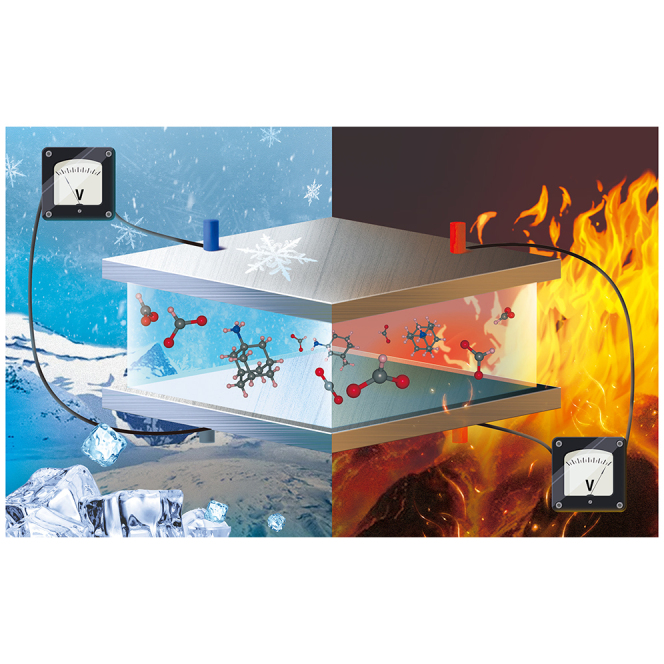

## Introduction

Pyroelectrics are polar materials whose electric polarizations can change with temperature. The ability to convert temperature changes into electrical signals allows them to be used in infrared detection, fire alarms, thermal imaging, and energy harvesting, etc.^1^^,^^2^ From a practical application point of view, the voltage figure of merit (FOM) *F*_v_ = *p*/*C*_v_*ε* is a particularly important parameter for gauging the electric voltage output efficiency, where *p* is the pyroelectric coefficient, *C*_v_ is the volume specific heat, and *ε* is the dielectric constant.^1^, ^2^, ^3^ Accordingly, high *F*_v_ values require large pyroelectric coefficients, small dielectric constants and heat capacities. Pyroelectrics are highly related to ferroelectrics in dipole polarizations; in fact, ferroelectrics are a class of pyroelectrics. In general, ferroelectrics have larger pyroelectric coefficients than non-ferroelectrics. For example, lead magnesium niobate-lead titanate (PMN-0.25PT), a traditional perovskite oxide ferroelectric, exhibits a large pyroelectric coefficient of *p* = −1,790 μC/m^2^·K, whereas non-ferroelectric aluminum nitride exhibits a small one, *p* = 6–8 μC/m^2^·K.^4^ This correlation also holds in the polar interfaces, as recently revealed by Yang et al.^5^ However, the dielectric constant of the PMN-0.25PT single crystal is large (*ε*_r_ = 2,100), leading to a low *F*_v_ value of 0.039 m^2^/C. Other inorganic ferroelectrics with large *p* usually behave similarly: large *p* and small *F*_v_.

In comparison, triglycine sulfate (TGS), an organic-inorganic hybrid ferroelectric, has a smaller pyroelectric coefficient (*p* = −280 μC/m^2^·K) and a considerably smaller dielectric constant (*ε*_r_ = 38).^2^ Its *F*_v_ value can reach 0.362 m^2^/C, which is nine times higher than that of PMN-PT. Thus, TGS has been a commercial pyroelectric material since the discovery of its pyroelectricity in the 1950s.^6^^,^^7^ Another example is an organic perrhenate hybrid [AH]ReO_4_ that exhibits an *F*_v_ value of about 0.45 m^2^/C at 298 K.^8^ The key to this high *F*_v_ can be attributed to the low *ε*_r_ and large *p*. In addition to these two hybrids, few pyroelectrics with such a high *F*_v_ value have been found. Over the past decade, organic-inorganic hybrids have been found as new ferroelectrics with properties comparable to their inorganic counterparts.^9^, ^10^, ^11^, ^12^ However, these reported organic-inorganic hybrid pyroelectrics suffer from either low Curie temperatures (*T*_C_) or small pyroelectric coefficients.

The relatively small dielectric constant of TGS is largely due to the weak polarizing ability under electric fields of the organic component glycine and the moderate ability of sulfate ions in the structure, similar to the case of [AH]ReO_4_. To further decrease the dielectric constant, all-organic ferroelectrics are good candidates for exploring pyroelectrics with better performance if their pyroelectric coefficients are sufficiently large. Moreover, organic materials are lightweight, flexible, and biocompatible, which are highly desired characteristics in the next generation of flexible devices.^13^, ^14^, ^15^, ^16^ However, to date, all-organic pyroelectrics have either low working temperatures or smaller *F*_v_s than TGS, considerably limiting their applications.^14^^,^^17^^,^^18^

It is known that molecules of various organic acids and amines are polar in structure. They can form simple organic salts, providing ample opportunities to find promising all-organic pyroelectrics. In TGS and other organic-inorganic hybrid pyroelectrics, the inorganic parts have negligible contributions to the total dipole polarizations in comparison with the organic parts. If both polar cations and polar anions are properly chosen in an organic salt, they are expected to simultaneously contribute to ferroelectricity and pyroelectricity. In addition, we expected the Curie temperature to be higher than room temperature within a range of several dozens of Kelvin and a continuous phase transition from ferroelectric to paraelectric with increasing temperature. This type of transition allows continuous change in the spontaneous polarization, instead of a sharp jump in the vicinity of the *T*_C_, and enables to maintain a large pyroelectric coefficient over a certain temperature range.

In this study, we initially chose the appropriate polar ions that are conducive to the emergence of ferroelectricity. As the smallest organic carboxylate ion, the formate anion is more likely to undergo an order-disorder transition in a crystal. Meanwhile, spherical-like cations with low rotational energy barriers are promising candidates for inducing structural phase transitions.^19^^,^^20^ Based on these considerations, a novel all-organic ferroelectric amantadine formate (AF) is found; AF is composed of two polar organic ions (Figure S1A) with *T*_C_ = 327 K, which is higher than that of TGS (322 K), but still close to room temperature. Structural analysis shows that both ions contributed to emergent ferroelectricity and pyroelectricity. Our measurements indicate that the ferroelectric-paraelectric phase transition in AF belongs to a continuous or second-order phase transition. As a result, the pyroelectric coefficient of AF is −170 μC/m^2^·K at 298 K. The room temperature dielectric constants of AF are only 13.5, 11.7, and 10.7, at 1, 10, and 100 kHz, respectively. The *F*_v_s are 0.705, 0.811, and 0.887 m^2^/C at the corresponding frequencies, respectively, higher than those of all known organics, and even TGS. In addition, the strain-electric field measurements reveal that the piezoelectric coefficient of AF is −16 pm/V, providing another example of the rare negative longitudinal piezoelectric effect.^21^, ^22^, ^23^, ^24^, ^25^ Our findings provide an all-organic material exhibiting high pyroelectric FOMs. AF has a low density of 1.21 g/cm^3^ and a low hardness (0.46 GPa). Moreover, it is non-toxic and inexpensive, both in raw materials and in synthesis. These traits make it a potential material for applications in flexible pyroelectric devices.

## Results and discussion

**Figure 1:**
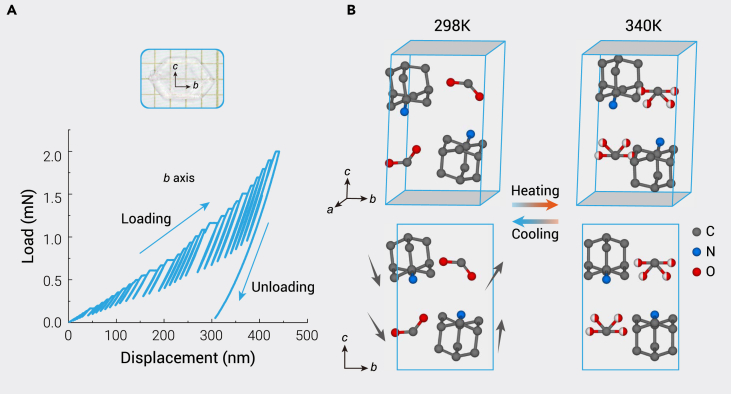
Mechanical properties and crystal structure of AF (A) Optical photograph of a grown single crystal of AF, and the load-displacement curve of AF single crystal along the *b* axis. The fitted elastic module and hardness are 8.74 GPa and 0.46 GPa, respectively. (B) Crystal structures of amantadine formate at 298 K (left) and 340 K (right), where the phase transition temperature is around 327 K. The arrows represent the directions of polarization of ions. The half red and half white balls represent the oxygen atoms with 50% occupation. Hydrogen atoms are omitted for clarity.

AF crystals up to 1 cm were grown by evaporating a mixed ethanol solution of amantadine and formic acid (Figures 1A and S1B). No other concomitant products were present, as confirmed by powder X-ray diffraction (Figure S2). Thermogravimetric analysis (TGA) indicates that AF is stable up to approximately 420 K (Figure S3). Although soluble in water, AF is very stable in air (Figure S4). The elastic module and hardness are measured to be 8.74 GPa and 0.46 GPa (Figure 1A), respectively, which are approximately one-third of the values for TGS (Figure S5) and one to two orders of magnitude smaller than the values for inorganic pyroelectrics.^26^ The crystal structures are determined from single-crystal X-ray diffraction (SCXRD) data (Tables S1–S3). AF crystallizes in a monoclinic system with space group *P*2_1_ at 298 K with cell parameters *a* = 8.2200(8) Å, *b* = 6.5851(7) Å, *c* = 10.4675(10) Å, *β* = 106.952(3)°, and *V* = 541.98(9) Å^3^. The calculated density is 1.21 g/cm^3^, slightly higher than that of water. The crystal structure is shown in Figure 1B. In the structure, a hydrogen atom of the formic acid molecule is ionized and acquired by the nitrogen atom of amantadine, forming two types of ions in the crystal: [C_10_H_18_N]^+^ and [HCOO]^–^. Each unit cell contains two AF molecules. There are several N–H···O hydrogen bonds with bond lengths from 2.771 Å to 2.795 Å connecting cations and anions. Both types of ions are polar, and the directions of the dipole moments are shown in Figure 1B. Thus, the net electric dipole moments of both [C_10_H_18_N]^+^ and [HCOO]^–^ arise along the *b* axis. The spontaneous polarization of AF can be expressed as Σ*p*_*i*_/*V* and is along the *b* axis, where *p*_*i*_ is the electric dipole moment of the ions and *V* is the volume.

We find that AF undergoes a structural phase transition with increasing temperature. At about 340 K, it recrystallizes in a centrosymmetric structure with space group *P*2_1_/*m*, followed by the disappearance of the spontaneous polarization. The cell becomes slightly larger: *a* = 8.2566(10) Å, *b* = 6.6287(6) Å, *c* = 10.4895(11) Å, *β* = 107.054(13)°, and *V* = 548.85(11) Å^3^. All [C_10_H_18_N]^+^ cations rotate a little and still remain ordered. Their net dipoles are lost as mirror planes that bisect the molecules now appear, as shown in Figure 1B. The [HCOO]^–^ anions also rotate and become disordered since they locate at two equivalent orientations connected by a mirror plane perpendicular to the *b* axis. Similarly, the net moment of the [HCOO]^–^ anions is also lost. The hydrogen bonds form from the H atoms of the amino groups and random O atoms of the formate anions. Hence, the bond lengths span a slightly larger range, from 2.725 Å to 2.828 Å. This phase transition between point group 2/*m* and 2 is one of the 88 species of ferroelectrics summarized by Aizu.^27^ A displacement of [C_10_H_18_N]^+^ ions and an order-disorder type change of [HCOO]^–^ ions occur when transiting from the ferroelectric to the paraelectric phase. Therefore, both contribute to polarization below the Curie temperature.

**Figure 2:**
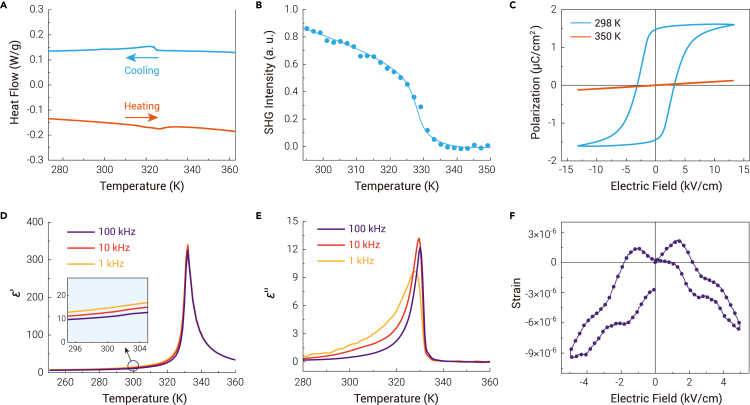
Ferroelectric-related properties (A) DSC data of AF during a heating and cooling cycle, revealing a phase transition around 327 K. (B) Temperature-dependent SHG intensity of polycrystalline sample of AF. (C) Polarization-electric field hysteresis loops along the *b* axis at 298 K and 350 K. The external electric field is a triangle wave with a frequency of 0.01 Hz. (D) Temperature-dependent real part of dielectric constants along the *b* axis at different frequencies. (E) Temperature-dependent imaginary part of dielectric constants along the *b* axis at different frequencies. (F) Strain-electric field hysteresis loop along the *b* axis at 298 K. The external electric field is a triangle wave with a frequency of 100 Hz.

To confirm this ferroelectric-paraelectric phase transition, we first detected the thermal response of AF. Differential scanning calorimetry (DSC) revealed that a peak appears at 327 K during the heating process, and at 322 K during the cooling process at a temperature change rate of 5 K/min (Figure 2A). Strikingly, in contrast to the *λ*-shaped peaks (Figure S6) observed in the phase transition for most ferroelectrics,^10^^,^^28^^,^^29^ the peaks of AF are step-like in shape, similar to ferroelectrics TGS,^7^ [C_8_H_9_N_2_]ClO_4_,^30^ and [(CH_3_)_3_NC_2_H_4_NH_3_]Pb_2_Cl_6_.^31^ This means that there is only a definite jump in specific heat rather than an indefinite divergence anomaly. Therefore, the phase transition in AF is second-order in nature. Variable-temperature second-harmonic generation (SHG) measurements were also performed to verify the structural phase transition (Figure 2B). Second-harmonic signals are only observed below 330 K, confirming that a phase transition from centrosymmetric to non-centrosymmetric occurs, consistent with the structure determined by SCXRD.

Temperature-dependent dielectric constant was measured on a single crystal. The real part of the dielectric constant (*ε*′) along the *b* axis shows *λ*-shaped anomalies around *T*_C_ (Figure 2D), similar to the imaginary part (*ε*′′, Figure 2E). However, *ε*′ is nearly temperature-independent along the *a* and *c* axes (Figure S7). The Curie-Weiss law is used to depict the behavior of *ε*′. It can be divided into two parts: *ε*′ = *C*_para_/(*T* – *T*_C_) for *T* > *T*_C_ and *ε*′ = *C*_ferro_/(*T*_C_ − *T*) for *T* < *T*_C_, where *C*_para_ and *C*_ferro_ are Curie-Weiss constants in the paraelectric and ferroelectric phases, respectively. As expected, the reciprocal value of *ε*′ for AF as a function of temperature is linear in both the paraelectric and ferroelectric phases, obeying the Curie-Weiss law (Figure S8). The ratio of *C*_para_/*C*_ferro_ for 1 kHz is 2.21, close to the theoretical value of 2 in second-order phase transition ferroelectric.^32^ This is again in good agreement with the DSC measurements. At 298 K, *ε*′ exhibited a slight frequency dependence (Figure S9A); the values at 1, 10, and 100 kHz are 13.5, 11.7, and 10.7, respectively, always smaller than those of TGS,^2^ whereas *ε*′′ is comparable to that of TGS, indicating the good insulation of AF (Figure S9B).

**Figure 3:**
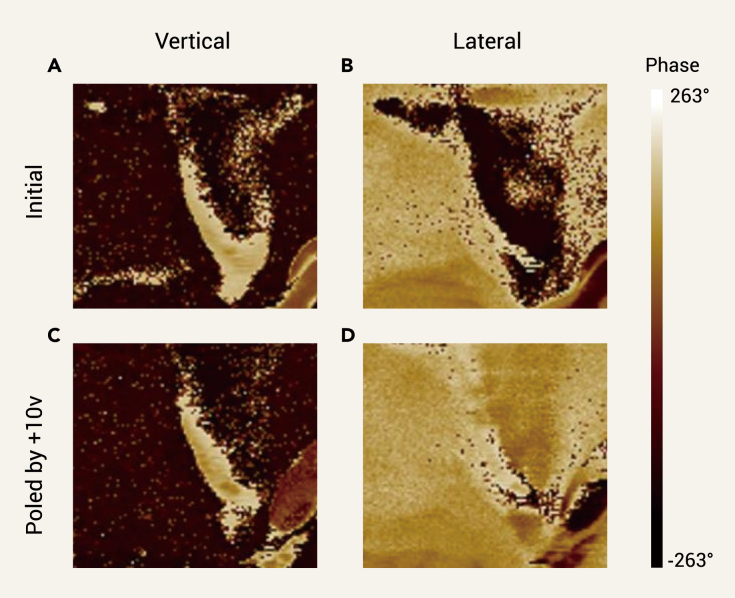
The PFM phase imaging before and after +10 V poling on a thin film of AF (A) The vertical phase before poling.(B) The lateral phase before poling.(C) The vertical phase after poling.(D) The lateral phase after poling. The area for observation and poling is 1 × 1 μm^2^. The measured temperature is room temperature.

One of the most important characteristics of ferroelectrics is the existence of polarization-electric (P-E) hysteresis loops. AF shows a standard P-E hysteresis loop for ferroelectricity at 298 K (Figure 2C). The remnant polarization is approximately 1.47 μC/cm^2^, which is larger than 0.25 μC/cm^2^ for typical molecular ferroelectric Rochelle salt,^13^ but smaller than 3.8 μC/cm^2^ for TGS.^33^ It should be noted that the polarization slowly decreases after removing the external electric field, indicating the existence of unstable domains. This may contribute to the dielectric dispersion at frequencies lower than 100 kHz.^34^^,^^35^ The coercive field is about 3.1 kV/cm, smaller than that of most molecular ferroelectrics.^13^^,^^36^, ^37^, ^38^ As poling is a necessary procedure for ferroelectrics before being used in pyroelectric devices, such a low coercive field will reduce the cost in the poling process. Meanwhile, the strain-electric field curve exhibits a typical butterfly shape of ferroelectrics and reveals that AF is a negative piezoelectric (Figures 2F and S10), which has attracted much interest recently.^21^, ^22^, ^23^, ^24^, ^25^^,^^39^^,^^40^ The estimated *d*_33_ is −16 pm/V, consistent with that measured by the Berlincourt method (Figure S11). The value is lower than −37.7 pm/V reported for negative-piezoelectric PVDF^22^ and −95 pm/V for CuInP_2_S_6_,^23^ but comparable to 6–16 pm/V for positive-piezoelectric LiNbO_3_,^41^ 14 pm/V for [C_7_H_16_N_2_]NH_4_I_3_,^42^ and 20 pm/V for [(CH_3_)_3_NOH]_2_KFe(CN)_6_.^43^ Furthermore, the structure and switching of domains are observed by piezoresponse force microscopy (PFM), strongly confirming the ferroelectricity of AF (Figure 3).

**Figure 4:**
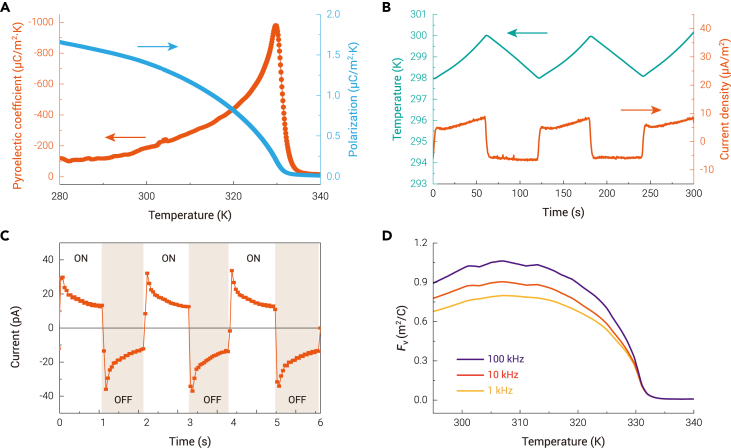
Pyroelectricity of AF (A) Temperature-dependent pyroelectric coefficient and polarization of AF. The latter is obtained by integrating the measured pyroelectric current over time. (B) Current responses under periodic temperature oscillations. (C) Current responses of AF under a periodically switched incandescent lamp. All the current measurements are along the *b* axis. (D) Temperature dependence of pyroelectric voltage FOM (*F*_v_).

The temperature dependence of the pyroelectric coefficient was obtained by measuring the current response under temperature ramping from 250 to 360 K. The pyroelectric coefficient first increases and reaches the maximum at *T*_C_. Beyond *T*_C_, it drops rapidly to zero, indicating the occurrence of a polar-nonpolar phase transition (Figure 4A). The polarizations at different temperatures were obtained by integrating the pyroelectric current density over time. At 298 K, the value is 1.44 μC/cm^2^, which is in good agreement with that obtained from the P-E hysteresis loop measurement. At temperatures far below the phase transition, the polarization decreases slowly with increasing temperature. When approaching the temperature of the phase transition, the rate of decrease gradually accelerates. As a result, large pyroelectric coefficients can exist in this temperature range (−118 μC/m^2^·K at 290 K to about −440 μC/m^2^·K at 320 K). We also measured the current response under periodic temperature oscillations around 298 K,^44^^,^^45^ and obtained a pyroelectric coefficient of approximately −170 μC/m^2^·K, which is close to the value obtained by temperature ramping. The corresponding pyroelectric current FOM is 0.83 × 10^−10^ m/V. Here, the volume specific heat measured by DSC is adopted in the calculation (Table S4). The room temperature pyroelectric coefficient of AF is comparable to that of perovskite oxides with polarization one order of magnitude larger than that of AF: LiTaO_3_ (−176 μC/m^2^·K),^1^ PbTiO_3_ (−180 μC/m^2^·K),^46^ and BaTiO_3_ (−200 μC/m^2^·K).^47^ This is due to the second-order phase transition and suitable *T*_C_. In ferroelectrics, both first- and second-order ferroelectric-paraelectric phase transitions provide large pyroelectric coefficients near *T*_C_. However, the polarization-temperature relationships of these two phase-transition types are different, resulting in different values of the pyroelectric coefficient. According to Landau theory,^32^ the order parameter *P*_r_ can be described by the following relations for the two types of phase transitions:

(Equation 1) $$\begin{array}{l} P_{r}={\left[\frac{-\beta +\sqrt{\beta^{2}-4\alpha_{0}\gamma \left(T-T_{c}\right)}}{2\gamma }\right]}^{1/2} \end{array}$$

and

(Equation 2) $$\begin{array}{l} P_{r}={\left[\frac{\alpha_{0}\left(T_{c}-T\right)}{\beta }\right]}^{1/2} \end{array}$$

where *α*_0_, *β*, and *γ* are constants. As shown in Figure S12, at temperatures *T* < *T*_C_, the second-order phase transition ferroelectric has a larger pyroelectric coefficient than the first-order one if they have the same remnant polarization. The temperature dependence of the polarization of AF agrees well with the ∼[*T*_C_ − *T*]^1/2^ law near *T*_C_ (Figure S13), confirming the second-order phase transition again. As an illustration, electrical currents are easily induced when irradiating an AF crystal by an incandescent lamp and vary synchronously with alternating switching (Figure 4C), confirming the sensibility to small changes in temperature.

**Figure 5:**
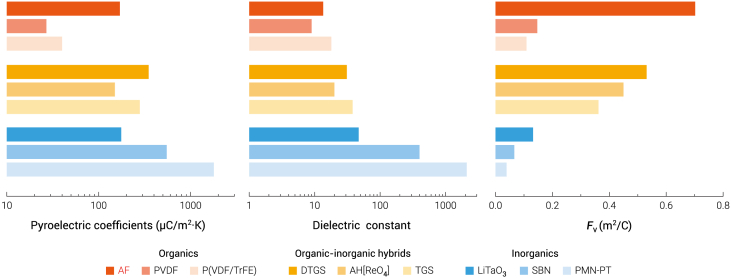
Comparison of pyroelectric coefficients, dielectric constants, and *F*_v_s between AF in this work and some famous pyroelectrics Where PVDF, P(VDF-TrFE), DTGS, SBN, and PMN-PT represent polyvinylidene fluoride, poly(50% vinylidene fluoride-50% trifluoroethylene), deuterated triglycine sulfate, Sr_0.5_Ba_0.5_Nb_2_O_6_, and 0.75Pb(Mg_1/3_Nb_2/3_)O_3_-0.25PbTiO_3_, respectively.^2^^,^^48^

Although those perovskite oxide ferroelectrics were found to have large pyroelectric coefficients, their *F*_v_ values are low (usually <0.1 m^2^/C) because of their large dielectric constants,^2^ whereas in AF, small dielectric constant and moderate volume specific heat (Figure S14) resulted in high values of *F*_v_ (Figure 4D). At 298 K, *F*_v_s are 0.705, 801, and 0.887 m^2^/C at 1, 10, and 100 kHz, respectively. These *F*_v_ values are considerably higher than those of most inorganic perovskites and even higher than that of TGS (Figure 5). In addition, other dielectric-constant-related pyroelectric FOMs of AF, such as detection capacity FOM (2.17 × 10^−5^ Pa^−1/2^) and energy harvesting FOM (5.94 × 10^−11^ m^3^/J), are comparable to those of commercial pyroelectric materials (Figure S15, Table S4).^49^ Thus, AF has a great potential to be used in highly sensitive thermal sensors.^2^ It should be noted that the secondary pyroelectric coefficients can be affected by piezoelectric behaviors through the expression *d*_*ijk*_*c*_*jklm*_*α*_*lm*_, where *d*, *c*, and *α* are piezoelectric, elastic, and thermal expansion coefficients, respectively.^50^ Here, a negative value of *d*_33_ and a positive value of *α*_33_ result in a negative component of secondary pyroelectric coefficient −2.8 μC/m^2^·K, where *c* = 8.74 GPa (Figure 1B) and *α* = 2 × 10^−5^ K^−1^ (Figure S16) are used for the calculation. The total pyroelectric coefficient is slightly enhanced because the primary and secondary coefficients have the same signs. But on the other side, this may cause noise in some applications. In the present case, the noise amplitude is about 1.6%, which is negligibly small. Moreover, the polarization of the negative piezoelectric was found to be abnormally enhanced under stress (Δ*P* = *d*_33_*σ*_33_, where *σ*_33_ is the stress).^39^ For AF, its polarization will nearly double under a stress of 1 GPa. The pyroelectric coefficient of AF is expected to be further enhanced under pressure.

## Conclusions

In summary, we discovered an all-organic ferroelectric, AF, which shows a high pyroelectric FOM around room temperature, where the *F*_v_ is higher than that of all known organic pyroelectrics, even TGS. A higher pyroelectric voltage output is expected in AF. The key to the high *F*_v_ is attributed to the continuous phase transition from ferroelectric to paraelectric and low dielectric constant. Meanwhile, AF is a new example for negative piezoelectric effect. Like TGS, the pyroelectric performance of AF can be further improved through molecular doping or molecular modification. Owing to the low density, low hardness, and low cost of this all-organic material, AF is expected to find a great potential to be applied in flexible pyroelectric devices.

## Materials and methods

The details of sample preparation and characterization are described in the supplemental information.

## Lead contact website

http://a02.iphy.ac.cn/

## Supplemental information

Document S1. Supplemental material and methods, Figures S1–S16, and Tables S1–S4

Document S2. Article plus Supplemental information
