# Supplementary material for: Discovery of amantadine formate: Toward achieving ultrahigh pyroelectric performances in organics
Source: Innovation (Camb). 2022 Jan 1;3(2):100204. doi: 10.1016/j.xinn.2021.100204 (PMC8803662; doi:10.1016/j.xinn.2021.100204)
Supplement: Document S1. Supplemental material and methods, Figures S1–S16, and Tables S1–S4 [file mmc1.pdf]

The Innovation, Volume *3*

Supplemental Information

Discovery of amantadine formate: Toward achieving ultrahigh pyroelectric performances in organics

Junyan Zhou, Shifeng Jin, Congcong Chai, Munan Hao, Xin Zhong, Tianping Ying, Jiangang Guo, and Xiaolong Chen

MATERIAL AND METHODS

Sample Preparations

In a typical run for amantadine formate (AF), 6 g of amantadine (Innochem, 98 wt. %) and 3 ml of formic acid (Innochem, 99 wt.%) were mixed into 50 ml anhydrous ethanol to get a clear and transparent solution. Dozens of crystals of AF with varying sizes were obtained by slowly evaporating the solvent at room temperature for about a week. Large, transparent and colorless crystals up to 1 cm were selected for property measurements. The crystals of triglycine sulfate (TGS) were preparation by slow cooling according to the literature.[1] Powder of barium titanate (99.9 wt. %) was purchased from Innochem. Ceramic of modified lead zirconate titanate (PIC 151) was purchased from Physik Instrumente.

The thin film of AF was grown by spin-coating method. The precursor solution is a saturated alcohol solution of AF. Then, 20 μL precursor solution was spread on a clean ITO-coated glass substrate. The thin films were obtained after spin-coating (1500 r/min) for 30 s and annealing at 313 K for 30 min.

Crystal structures determination

Single crystal diffraction data were collected using a Bruker D8 VENTURE PHOTO II diffractometer and a Rigaku XtaLAB Synergy R diffractometer for 298 K and 340 K, respectively. Both of the sources were multilayer mirror monochromatized Mo Kα (λ = 0.71073 Å) radiation. Data collection, cell refinement, and data reduction were carried out in the Bruker APEX program for 298 K and Rigaku CrysAlis PRO program for 340 K. The structures were solved by direct methods and refined by the full-matrix method based on F^2 using the SHELXTL software package.[2] All non-hydrogen atoms were refined anisotropically, and the H atoms were placed in geometrically idealized positions. Experimental details are listed in Table S1. CCDC 2077688 and 2077689 contain the supplementary crystallographic data for this paper. These data can be obtained free of charge from The Cambridge Crystallographic Data Centre via www.ccdc.cam.ac.uk/data_request/cif.

Variable temperature PXRD measurements were performed on a Rigaku SmartLab diffractometer with Cu Kα radiation (λ = 1.5406 Å, 40 kV, 30 mA) and a graphite monochromator in a reflection mode (2θ = 5° to 80°, step = 0.01 °2θ, and scan speed = 1 °·min^{-1}). Indexing and Rietveld refinements were performed using the DICVOL91 and FULLPROF programs, respectively.[3,4]

Thermal properties

Thermogravimetric analyses (TGA) were carried out using TA SDTQ600 thermal analyzer under Ar atmosphere in the temperature range of 20−350 °C in alumina crucibles with a heating rate of 10

K·min^{-1}. Differential scanning calorimetry (DSC) measurements were carried out using a TA DSCQ200 thermal analyzer under Ar atmosphere with heating and cooling rates of 5 K·min^{-1}.

Elastic modules and hardnesses

Nano-indentation measurements were carried out using Bruker TI 980 triboIndenter. TGS was used to compare with AF, the samples are both single crystals along the *b* axis. The measured elastic modules of TGS and AF are 24.18 GPa and 8.74 GPa, respectively. The fitted hardnesses of TGS and AF are 1.30 GPa and 0.46 GPa, respectively.

Second harmonic generations

The second harmonic generations (SHG) measurements were employed by EKSPLA PL2210A laser beam with low divergence (pulsed Nd:YAG, λ = 1064 nm). The powder of AF was put into a UV cuvette. The reflected light of the sample is received by a spectrometer, and the SHG intensity was obtained from the reflected light intensity around 532 nm. The temperature was controlled by HFS600E LinKam stage.

Dielectric constants

The dielectric constant measurements were employed by Keysight impedance analyzer with an applied ac electric field of 500 mV. Crystals of AF and TGS were cut into a plate perpendicular to the *b* axis, the sizes are 2.0 mm^2 × 0.32 mm for AF and 3.1 mm^2 × 0.32 mm for TGS. Silver paste was coated as electrodes on both sides. The temperature was also controlled by HFS600E LinKam stage.

P-E hysteresis loops

The polarization-electric field (P-E) measurements were performed on aixACCT TF Analyzer 3000 Measurement System. The sample was the same one for dielectric constant measurements. The temperature was controlled by Delta 9023 oven.

Piezoelectricity

We used two methods to measure the piezoelectric coefficients: interferometer and Berlincourt methods. The interferometer method is performed on aixACCT TF Analyzer 3000 Measurement System. The piezoelectric coefficient d_{33} was estimated by measuring the strain-electric field loop. The Berlincourt method is carried out using a ZJ-3AN quasi-static d_{33} meter, the gear position is "×0.1".

Piezoresponse force microscopy measurements

The PFM measurement was carried out on Bruker Nano Inc. atomic force microscope at room temperature. The frequencies of AC drive voltage for both out-plane and in-plane phase imaging were 350 kHz.

Pyroelectricity

The sample for pyroelectric measurements was same for dielectric constant measurements. Before the measurements, it was poled by an electric field of 10 $kV \cdot cm^{-1}$ (coercive field is about 3.1 $kV \cdot cm^{-1}$) for 100 s. The pyroelectric coefficients from 250~350 K were measured by temperature ramping techniques [5]. The sample was firstly cooled to 200 K, After that, it was heated to 360 K with a rate of 2 $K \cdot min^{-1}$. The pyroelectric coefficient equals the pyroelectric current density divided by the heating rate. In addition, periodic temperature change techniques [5] was performed at 298 K. The sample was in a temperature oscillation in the form of triangular wave with amplitude of 2 K and period of 120 s. The current was recorded by aixACCT TF Analyzer 3000 Measurement System, and the temperature was controlled by HFS600E LinKam stage.

The sample for measuring the electric response to surface illumination was same for pyroelectric measurements. A incandescent lamp was used to generating periodically illumination. The illumination area was much larger than the sample size.

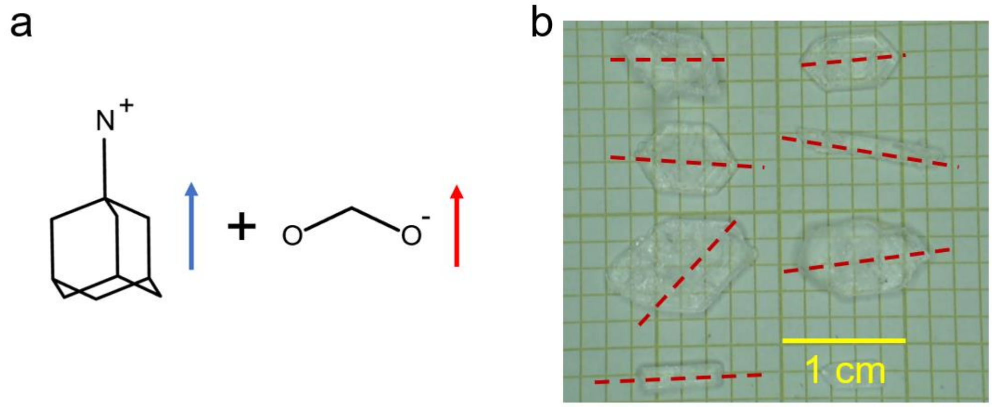

Fig. S1 a, Schematic structures of amantadine (left) and formate (right) ions in AF. The bond types of C-O are not distinguished in formate. Both ions are polar and the directions of their dipole moments are represented by blue and red arrows. **b,** Optical photograph of the grown single crystals of AF. The red dashed lines represent the *b* axis of the crystals, and the *c* axis is perpendicular to the grid paper.

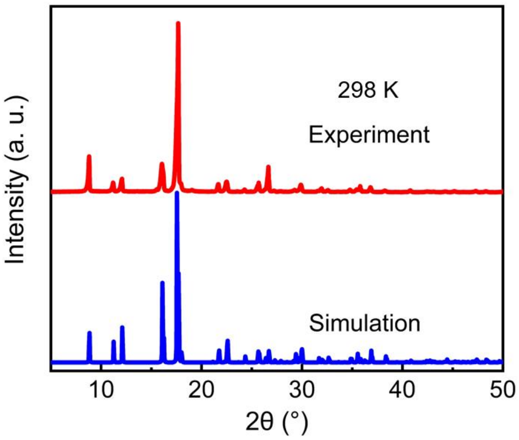

Fig. S2 Powder X-ray diffraction pattern of amantadine formate at 298 K, the blue curve is simulated from the structure obtained by single crystal X-ray diffraction.

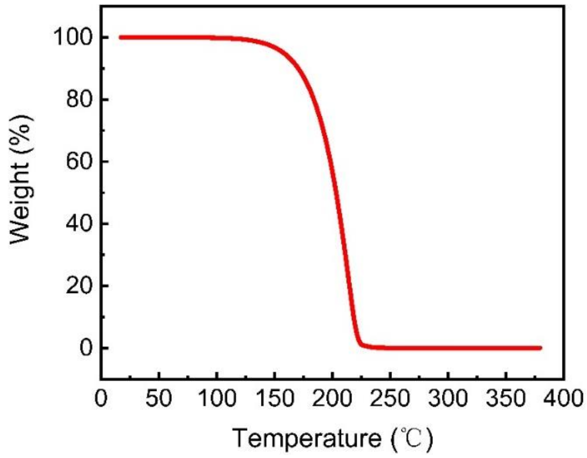

Fig. S3 Thermogravimetry of AF, the decomposition (or sublimation) temperature is about 420 K.

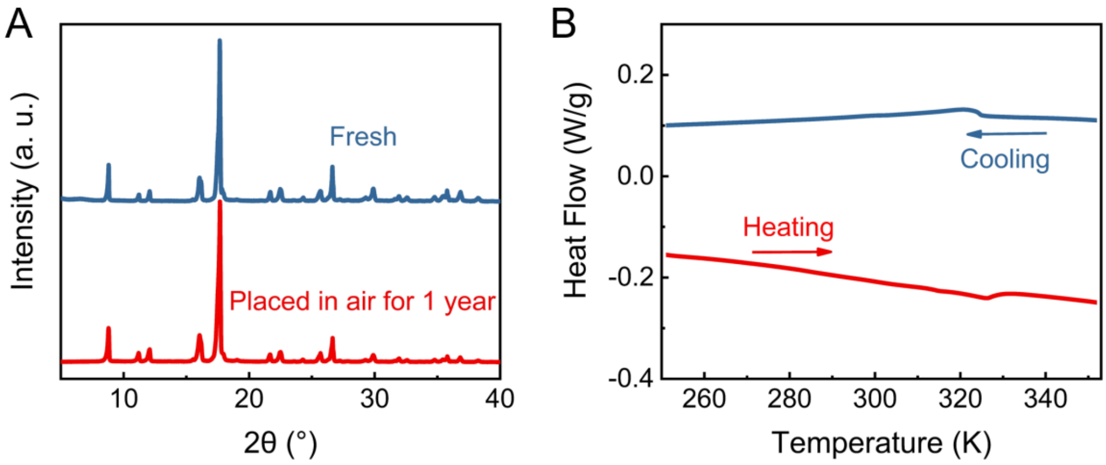

Fig. S4 (A) Comparation of PXRD patterns between AF powder just synthesized and that placed for about one year in air. (B) DSC curves of AF placed for about one year in air. The sample was firstly cooled to 240 K. The heat flow was measured during a 240 K-360 K-240 K cycle in a temperature changing rate of 5 K/min. There is no themal signal of water melting or freezing.

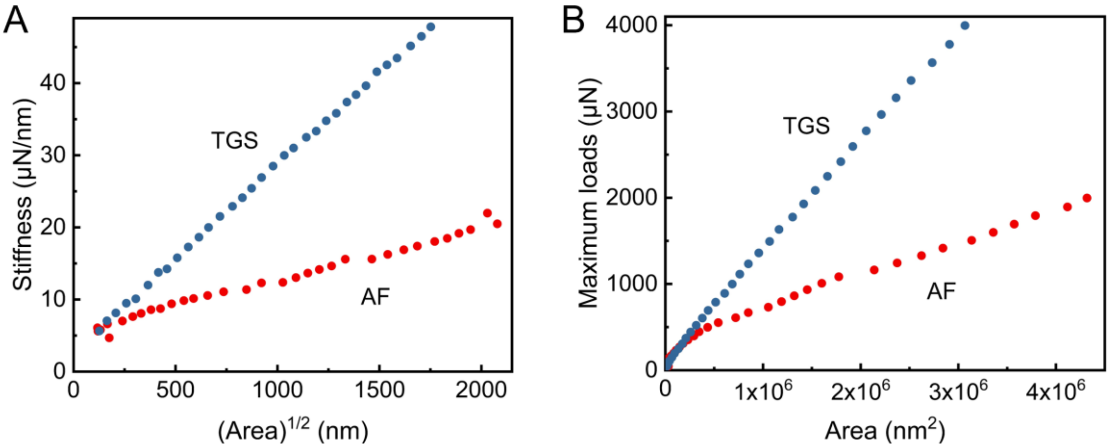

Fig. S5 Results of nano-indentation measurements on single crystals of both TGS and AF. (A) Stiffnesses as functions of the square root of indentation area. The fitted elastic modules of TGS and AF are 24.18 GPa and 8.74 GPa, respectively. (B) Maximum loads as functions of indentation area. The fitted hardnesses of TGS and AF are 1.30 GPa and 0.46 GPa, respectively. Both are measured along the *b* axis.

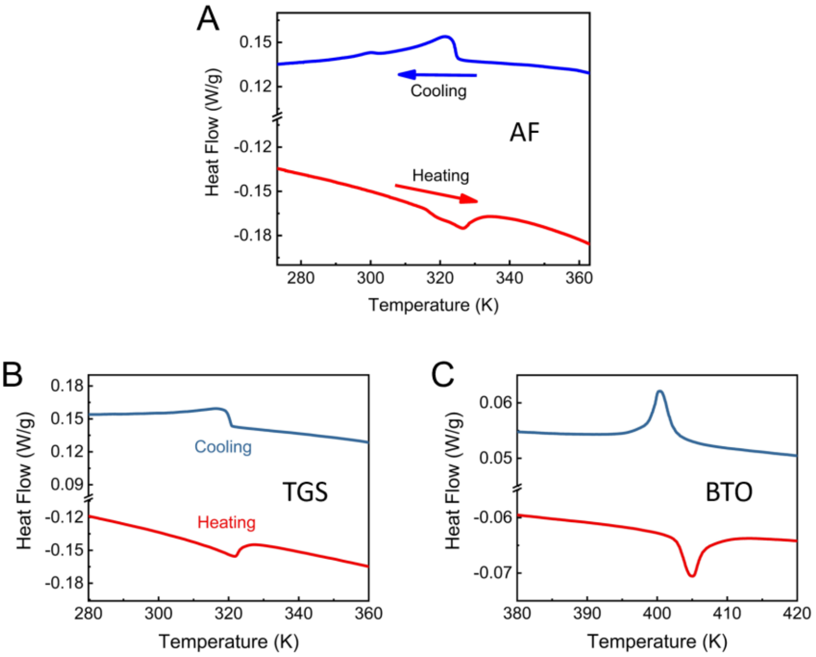

Fig. S6 DSC curves of ferroelectrics. (A) AF in this work. (B) TGS. (C) $BaTiO_3$. Where $BaTiO_3$ and TGS are well-known ferroelectrics with first-order phase transition and second-order phase transition, respectively.

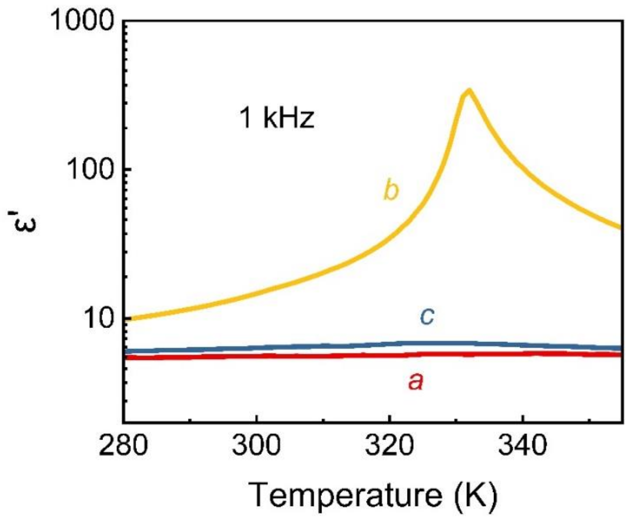

Fig. S7 Anisotropic real part of dielectric constants of AF.

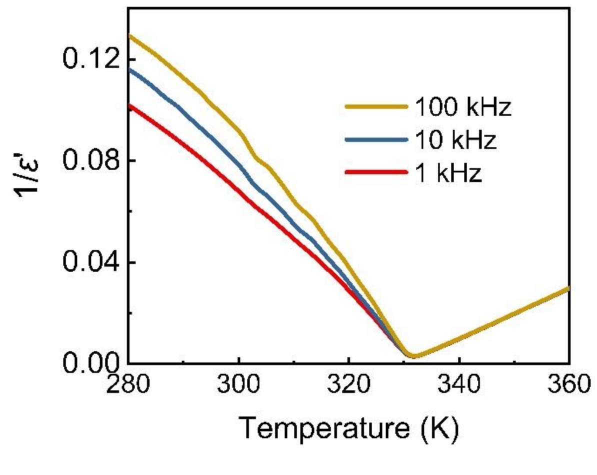

Fig. S8 Temperature dependence of the reciprocal of dielectric constants for AF at several frequencies.

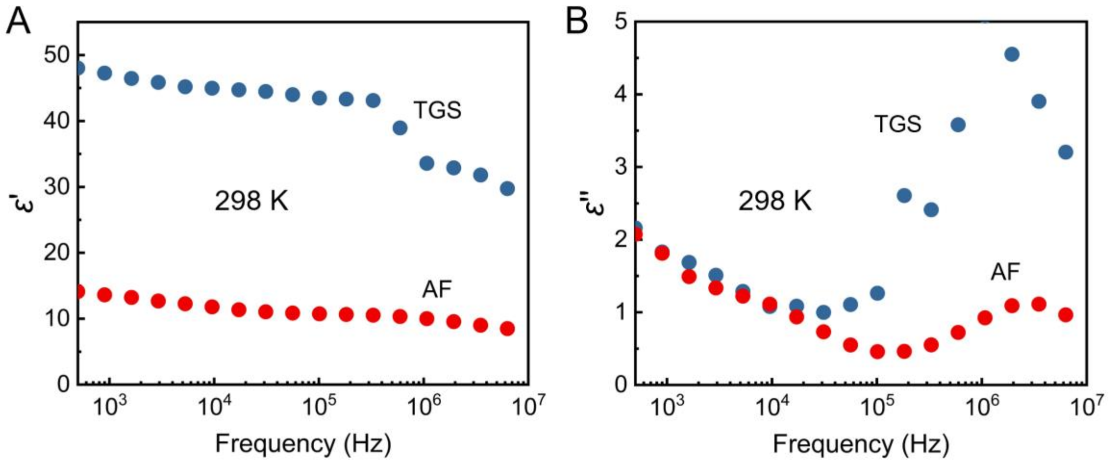

Fig. S9 Dispersion of complex dielectric constants at 298 K for TGS and AF (A) Real part. (B) Imaginary part. Both are measured along the *b* axis.

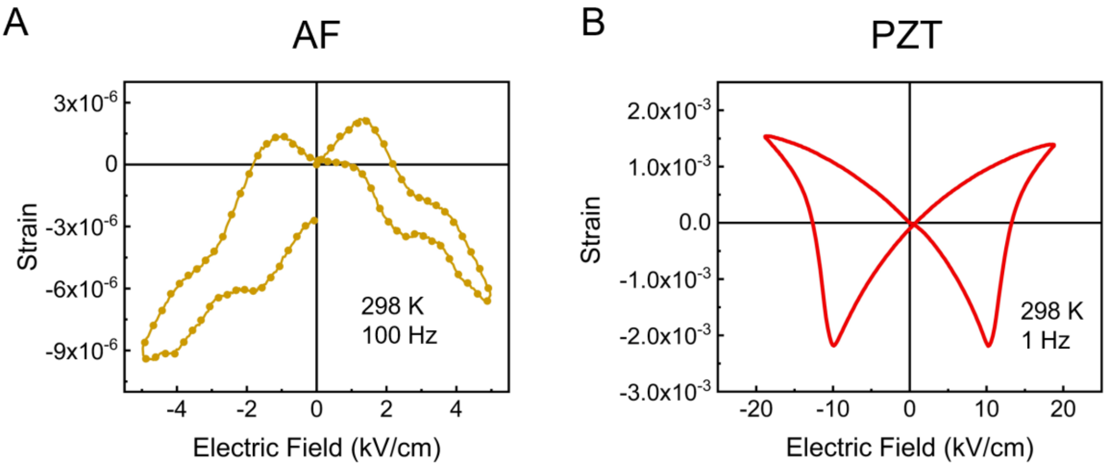

Fig. S10 Strain-electric field curves of (A) AF (*b* axis) in this work and (B) modified PZT. The latter is a positive piezoelectric. The positive value of strain represents elongation, while negative for shortening. The estimated piezoelectric coefficients are −16 pm·V^{-1} for AF and 797 pm·V^{-1} for modified PZT. The measured temperature is 298 K, the frequencies are 100 Hz for AF and 1 Hz for modified PZT.

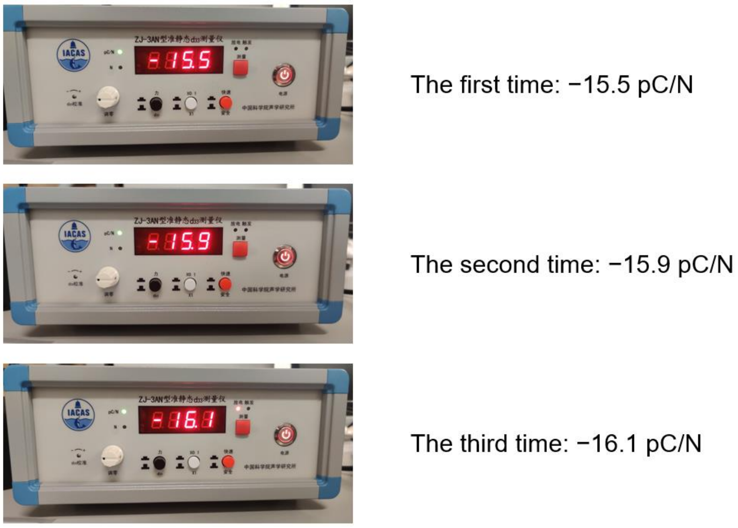

Fig. S11 Results of d_{33} measured by Berlincourt methods, giving a d_{33} value −15.8 ± 0.5 pC/N.

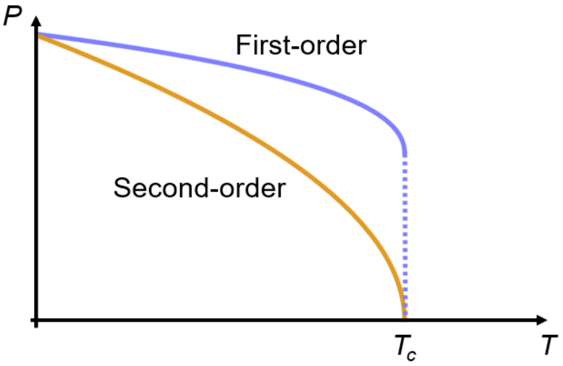

Fig. S12 Comparation of the polarization-temperature behavior of first-order and second-order ferroelectric-paraelectric phase transition based on Landau theory.

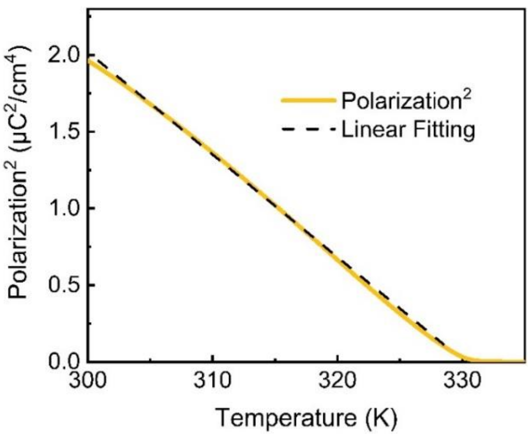

Fig. S13 The relationship between polarization square of AF and temperature. The curve from 300 K to 330 K is well fitted by a straight line, suggests the ~ $[T_C - T]^{1/2}$ law of polarization near T_C.

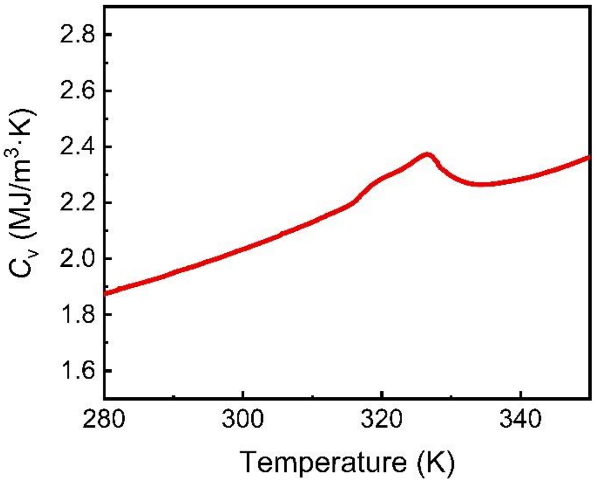

Fig. S14 Temperature-dependent volume specific heat of AF, calculated from the heating run of DSC curve.

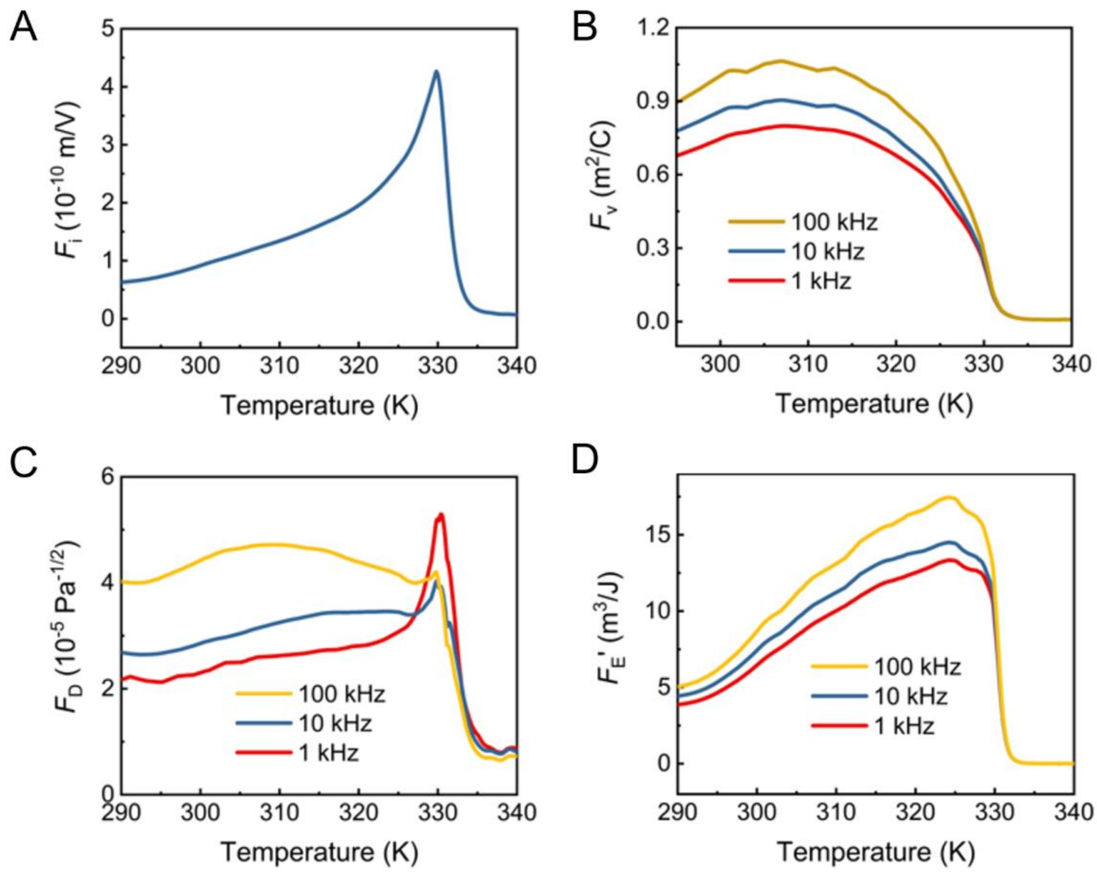

Fig. S15 Temperature-dependent pyroelectric FOMs for current sensitivity (A), voltage responsivity (B), detection capacity (C), and energy harvesting (D).

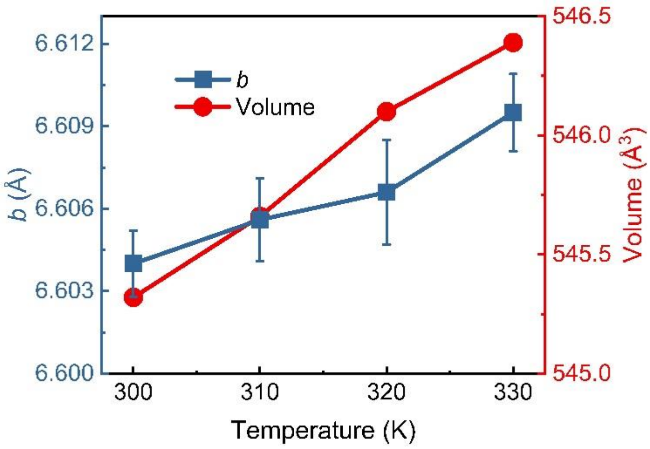

Fig. S16 Temperature dependence of the cell parameter *b* and the unit cell volume of AF. The thermal expansion coefficient along the *b*-axis is estimated to be 2×10^{-5} K^{-1}.

Table S1. Crystal Data of AF at Different Temperatures

Formula	$[C_{10}H_{18}N][HCOO]$	
Formula weight	197.28	
Temperature	298 K	340 K
Crystal system	Monoclinic	
Space group	$P2_1$	$P2_1/m$
a (Å)	8.2200(8)	8.2566(10)
b (Å)	6.5851(7)	6.6287(6)
c (Å)	10.4675(10)	10.4895(11)
β (°)	106.952(3)	107.054(13)
Volume (Å^3)	541.98(9)	548.85(11)
Z	2	2
Density (g·cm^{-3})	1.21	1.19
F(000)	242.0	242.0
θ range for data collection (°)	2.034-28.384	2.580-30.458
Index ranges	$-10 \leq h \leq 10$<br>$-8 \leq k \leq 8$<br>$-13 \leq l \leq 14$	$-10 \leq h \leq 11$<br>$-9 \leq k \leq 8$<br>$-13 \leq l \leq 9$
Independent reflections	2694	1570
Goodness-of-fit on F^2	1.055	0.980
R_1 [$I > 2\sigma(I)$]	0.0574	0.0638
wR_2 [$I > 2\sigma(I)$]	0.1583	0.1876

$R_1 = \sum||F_o| - |F_c||/\sum|F_o|$, $wR_2 = \{\sum[w(|F_o|^2 - |F_c|^2)]/\sum[w|F_o|^4]\}^{1/2}$

Table S2. Fractional atomic coordinates, equivalent isotropic displacement parameters and occupancy rates for non-hydrogen atoms in AF at 298 K.

Atom	*x*	*y*	*z*	U_{iso}	Occupancy rate
C1	0.8696(3)	0.1531(3)	0.6888(2)	0.0392(4)	1
C2	0.9520(3)	0.1290(4)	0.8386(2)	0.0483(6)	1
C3	0.7558(3)	-0.0271(4)	0.6331(3)	0.0489(6)	1
C4	0.7654(3)	0.3494(3)	0.6624(3)	0.0478(6)	1
C5	0.6249(4)	0.3361(4)	0.7308(3)	0.0547(6)	1
C6	0.6162(4)	-0.0402(4)	0.7024(3)	0.0551(7)	1
C7	0.8117(4)	0.1147(4)	0.9072(2)	0.0559(7)	1
C8	0.6983(5)	-0.0662(5)	0.8530(3)	0.0637(8)	1
C9	0.7061(4)	0.3084(5)	0.8811(3)	0.0624(7)	1
C10	0.5110(3)	0.1530(5)	0.6767(3)	0.0615(7)	1
C11	0.2227(3)	0.6670(4)	0.7361(2)	0.0468(5)	1
N1	1.0073(3)	0.1666(3)	0.6212(2)	0.0483(5)	1
O1	0.2190(3)	0.4822(4)	0.7429(3)	0.0810(8)	1
O2	0.1468(4)	0.7766(4)	0.6427(2)	0.0736(7)	1

Table S3. Fractional atomic coordinates, equivalent isotropic displacement parameters and occupancy rates for non-hydrogen atoms in AF at 340 K.

Atom	*x*	*y*	*z*	U_{iso}	Occupancy rate
C1	0.3701(3)	0.2500	0.6888(2)	0.0604(6)	1
C2	0.2616(2)	0.0622(3)	0.64769(18)	0.0759(6)	1
C3	0.1229(2)	0.0635(3)	0.71688(19)	0.0832(6)	1
C4	0.0135(3)	0.2500	0.6760(3)	0.0891(9)	1
C5	0.3133(4)	0.2500	0.9070(2)	0.0884(9)	1
C6	0.2039(3)	0.0639(3)	0.8669(2)	0.0979(8)	1
C7	0.4524(3)	0.2500	0.8386(2)	0.0729(7)	1
C8	0.2773(3)	0.2500	0.2658(2)	0.0731(8)	1
N1	0.5072(3)	0.2500	0.62081(19)	0.0754(7)	1
O1	0.3569(4)	0.1277(5)	0.3568(3)	0.0986(11)	0.5
O2	0.2800(4)	0.0775(5)	0.2651(4)	0.1070(11)	0.5

Table S4. Comparison of room-temperature pyroelectric-related properties between AF and other famous pyroelectrics. Where $F_i = p/C_v$, $F_v = p/\varepsilon' C_v$, $F_D = p/C_v(\varepsilon'')^{1/2}$, $F_E' = p^2/\varepsilon'(C_v)^2$.

	Materials	p μC/m²·K	C_v MJ/m³·K	ε'	ε''	F_i 10^{-10} m/V	F_v m²/C	F_D 10^{-5} $Pa^{-1/2}$	F_E' 10^{-11} m³/J
Organics	**AF This Work**	**170**	**2.02**	**13.5 (1 kHz)**	**1.7 (1 kHz)**	**0.843**	**0.705**	**2.17**	**5.94**
	PVDF[6]	52	2.2	18	0.95	0.24	0.146	0.80	0.35
	P(VDF/TrFE) 90/10[6]	80	2.6	22	0.90	0.31	0.159	1.1	0.49
Organic-inorganic hybrids	DTGS[7]	400	2.4	33	0.66	1.67	0.57	6.9	9.52
	$AH[ReO_4]$[8]	150	1.84	20.5	0.5	0.82	0.45	3.84	3.69
	TGS[7]	350	2.6	40	1.0	1.35	0.38	4.5	5.13
Inorganics	$LaTiO_3$[9]	190	3.2	47	0.24	0.59	0.14	4.1	0.83
	SBN[9]	550	2.2	400	1.2	2.48	0.07	7.2	1.74
	PMN-0.13PT[10]	3260	2.6	3107	10.6	13.0	0.046	13.5	5.98

References

1. Senthil Pandian, M., Verma, S., Karuppasamy, P., et al. (2020). TGS crystal growth below and above Curie temperature (Tc). J. Cryst. Growth ***546***, 125793, 10.1016/j.jcrysgro.2020.125793.
2. Sheldrick, G.M. (2008). A short history of SHELX. Acta Crystallogr. A ***64***, 112-122, 10.1107/S0108767307043930.
3. Louër, D., and Louër, M. (1972). Méthode d'essais et erreurs pour l'indexation automatique des diagrammes de poudre. J. Appl. Crystallogr. ***5***, 271-275, 10.1107/s0021889872009483.
4. Rodríguez-Carvajal, J. (1993). Recent advances in magnetic structure determination by neutron powder diffraction. Physica B ***192***, 55-69, 10.1016/0921-4526(93)90108-i.
5. Lubomirsky, I., and Stafsudd, O. (2012). Invited review article: practical guide for pyroelectric measurements. Rev. Sci. Instrum. ***83***, 051101, 10.1063/1.4709621.
6. Dietze, M., Krause, J., Solterbeck, C.H., and Es-Souni, M. (2007). Thick film polymer-ceramic composites for pyroelectric applications. J. Appl. Phys. ***101***, 054113, 10.1063/1.2653978.
7. Felix, P., Gamot, P., Lacheau, P., and Raverdy, Y. (1977). Pyroelectric, dielectric and thermal properties of TGS, DTGS and TGFB. Ferroelectrics ***17***, 543-551, 10.1080/00150197808236779.
8. Harada, J., Kawamura, Y., Takahashi, Y., et al. (2019). Plastic/Ferroelectric Crystals with Easily Switchable Polarization: Low-Voltage Operation, Unprecedentedly High Pyroelectric Performance, and Large Piezoelectric Effect in Polycrystalline Forms. J. Am. Chem. Soc. ***141***, 9349-9357, 10.1021/jacs.9b03369.
9. Whatmore, R.W., Patel, A., Shorrocks, N.M., and Ainger, F.W. (1990). Ferroelectric materials for thermal ir sensors state-of-the-art and perspectives. Ferroelectrics ***104***, 269-283, 10.1080/00150199008223829.
10. Yu, P., Tang, Y., and Luo, H. (2007). Fabrication, property and application of novel pyroelectric single crystals—PMN–PT. J. Electroceram. ***24***, 1-4, 10.1007/s10832-007-9360-7.
