# Supplementary material for: Discovery of amantadine formate: Toward achieving ultrahigh pyroelectric performances in organics
Source: Innovation (Camb). 2022 Jan 1;3(2):100204. doi: 10.1016/j.xinn.2021.100204 (PMC8803662; doi:10.1016/j.xinn.2021.100204)
Supplement: Document S2. Article plus Supplemental information [file mmc2.pdf]

Report

Discovery of amantadine formate: Toward achieving ultrahigh pyroelectric performances in organics

Junyan Zhou,[1,2] Shifeng Jin,[1,2,*] Congcong Chai,[1,3] Munan Hao,[1,2] Xin Zhong,[1] Tianping Ying,[4] Jiangang Guo,[1,5] and Xiaolong Chen[1,2,5,*]
*Correspondence: shifengjin@iphy.ac.cn (S.J.); chenx29@iphy.ac.cn (X.C.)
Received: September 22, 2021; Accepted: December 30, 2021; Published Online: January 1, 2022; https://doi.org/10.1016/j.xinn.2021.100204
© 2022 The Authors. This is an open access article under the CC BY license (http://creativecommons.org/licenses/by/4.0/).

GRAPHICAL ABSTRACT

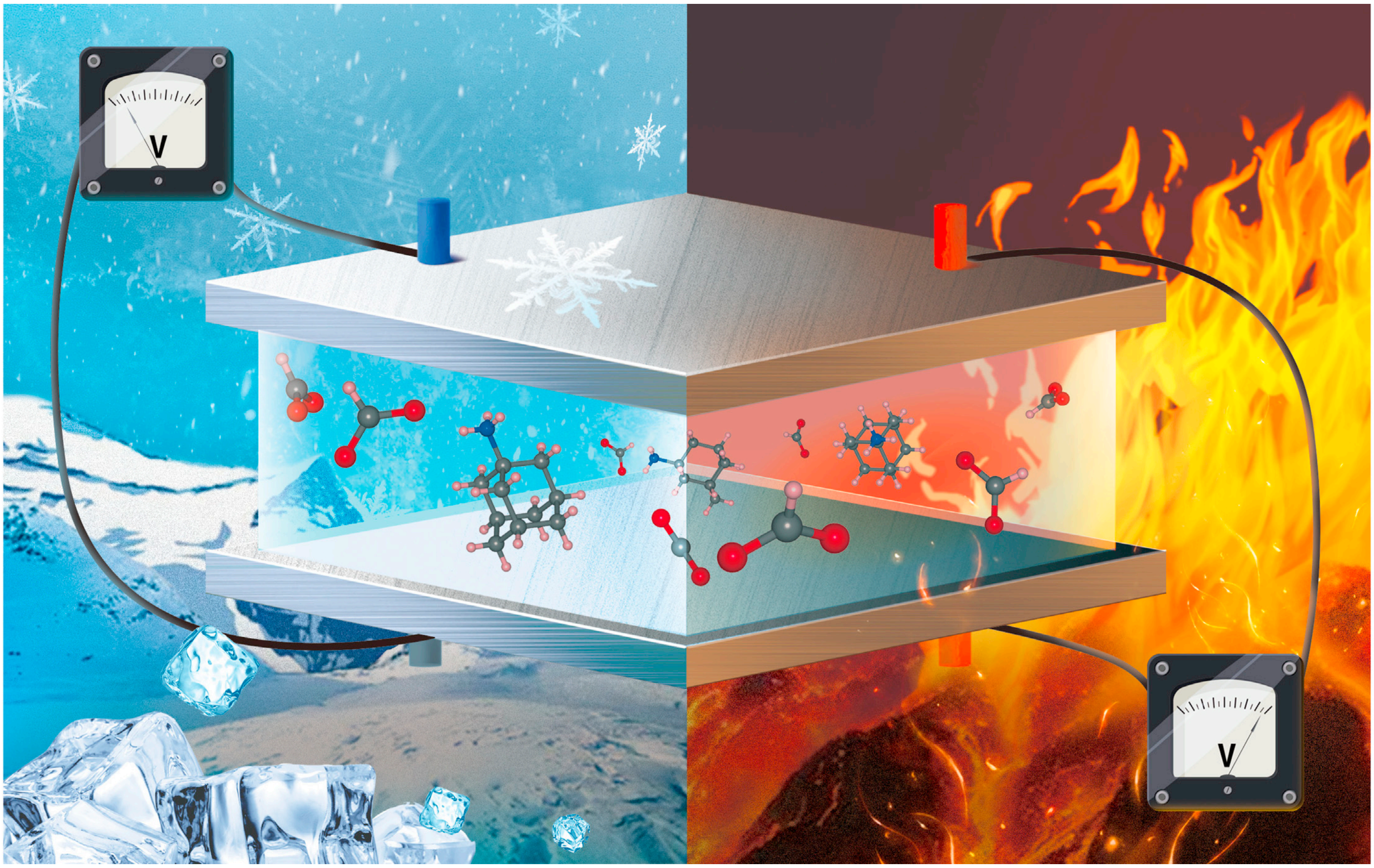

PUBLIC SUMMARY

- Organic pyroelectrics have great potential in wearable devices for temperature sensing, IR detection, thermal imaging, and energy harvesting
- We report the first all-organic pyroelectric amantadine formate with properties better than that of TGS, a hybrid pyroelectric in use since the 1950s
- Amantadine formate has a large pyroelectric coefficient and a surprisingly small dielectric constant, which play a key role in its excellent pyroelectric performance
- The strategy of combining all-organic components and second-order phase transition will contribute to the exploration of new pyroelectrics

Report

The Innovation

Discovery of amantadine formate: Toward achieving ultrahigh pyroelectric performances in organics

Junyan Zhou,[1,2] Shifeng Jin,[1,2,*] Congcong Chai,[1,3] Munan Hao,[1,2] Xin Zhong,[1] Tianping Ying,[4] Jiangang Guo,[1,5] and Xiaolong Chen[1,2,5,*]

[1]Beijing National Laboratory for Condensed Matter Physics, Institute of Physics, Chinese Academy of Sciences, Beijing 100190, China

[2]School of Physical Sciences, University of Chinese Academy of Sciences, Beijing 101408, China

[3]College of Materials Science and Opto-Electronic Technology, University of Chinese Academy of Sciences, Beijing 101408, China

[4]Materials Research Center for Element Strategy, Tokyo Institute of Technology, Yokohama 226-8503, Japan

[5]Songshan Lake Materials Laboratory, Dongguan 523808, China

*Correspondence: shifengjin@iphy.ac.cn (S.J.); chenx29@iphy.ac.cn (X.C.)

Received: September 22, 2021; Accepted: December 30, 2021; Published Online: January 1, 2022; https://doi.org/10.1016/j.xinn.2021.100204

© 2022 The Authors. This is an open access article under the CC BY license (http://creativecommons.org/licenses/by/4.0/).

Citation: Zhou J., Jin S., Chai C., et al., (2022). Discovery of amantadine formate: Toward achieving ultrahigh pyroelectric performances in organics. The Innovation **3(2)**, 100204.

Pyroelectrics are a class of polar compounds that output electrical signals upon changes in temperature. With the rapid development of flexible electronics, organic pyroelectrics are highly desired. However, most organics suffer from low pyroelectric coefficients or low working temperatures. To date, the realization of superior pyroelectric performance in all-organics has remained a challenge. Here, we report the discovery of amantadine formate, an all-organic pyroelectric with ultrahigh voltage figures of merit (F_v), surpassing those of all other known organics and commercial triglycine sulfate, $LiTaO_3$ as well around room temperature. The key to the high F_v is attributed to large pyroelectric coefficients in a favorable temperature range resulting from a ferroelectric-paraelectric phase transition of second order at 327 K, small dielectric constant, and moderate heat capacity. In addition, amantadine formate is relatively lightweight, soft, transparent, low-cost, and non-toxic, adding value to its potential applications in flexible electronics. Our results demonstrate that a new type of pyroelectrics can exist in organic compounds.

INTRODUCTION

Pyroelectrics are polar materials whose electric polarizations can change with temperature. The ability to convert temperature changes into electrical signals allows them to be used in infrared detection, fire alarms, thermal imaging, and energy harvesting, etc.[1,2] From a practical application point of view, the voltage figure of merit (FOM) $F_v = p/C_v\epsilon$ is a particularly important parameter for gauging the electric voltage output efficiency, where p is the pyroelectric coefficient, C_v is the volume specific heat, and ϵ is the dielectric constant.[1-3] Accordingly, high F_v values require large pyroelectric coefficients, small dielectric constants and heat capacities. Pyroelectrics are highly related to ferroelectrics in dipole polarizations; in fact, ferroelectrics are a class of pyroelectrics. In general, ferroelectrics have larger pyroelectric coefficients than non-ferroelectrics. For example, lead magnesium niobate-lead titanate (PMN-0.25PT), a traditional perovskite oxide ferroelectric, exhibits a large pyroelectric coefficient of $p = -1{,}790\ \mu C/m^2\cdot K$, whereas non-ferroelectric aluminum nitride exhibits a small one, $p = 6–8\ \mu C/m^2\cdot K$.[4] This correlation also holds in the polar interfaces, as recently revealed by Yang et al.[5] However, the dielectric constant of the PMN-0.25PT single crystal is large ($\epsilon_r = 2{,}100$), leading to a low F_v value of 0.039 m^2/C. Other inorganic ferroelectrics with large p usually behave similarly: large p and small F_v.

In comparison, triglycine sulfate (TGS), an organic-inorganic hybrid ferroelectric, has a smaller pyroelectric coefficient ($p = -280\ \mu C/m^2\cdot K$) and a considerably smaller dielectric constant ($\epsilon_r = 38$).[2] Its F_v value can reach 0.362 m^2/C, which is nine times higher than that of PMN-PT. Thus, TGS has been a commercial pyroelectric material since the discovery of its pyroelectricity in the 1950s.[6,7] Another example is an organic perrhenate hybrid [AH]ReO_4 that exhibits an F_v value of about 0.45 m^2/C at 298 K.[8] The key to this high F_v can be attributed to the low ϵ_r and large p. In addition to these two hybrids, few pyroelectrics with such a high F_v value have been found. Over the past decade, organic-inorganic hybrids have been found as new ferroelectrics with properties comparable to their inorganic counterparts.[9–12] However, these reported organic-inorganic hybrid pyroelectrics suffer from either low Curie temperatures (T_C) or small pyroelectric coefficients.

The relatively small dielectric constant of TGS is largely due to the weak polarizing ability under electric fields of the organic component glycine and the moderate ability of sulfate ions in the structure, similar to the case of [AH]ReO_4. To further decrease the dielectric constant, all-organic ferroelectrics are good candidates for exploring pyroelectrics with better performance if their pyroelectric coefficients are sufficiently large. Moreover, organic materials are lightweight, flexible, and biocompatible, which are highly desired characteristics in the next generation of flexible devices.[13–16] However, to date, all-organic pyroelectrics have either low working temperatures or smaller F_vs than TGS, considerably limiting their applications.[14,17,18]

It is known that molecules of various organic acids and amines are polar in structure. They can form simple organic salts, providing ample opportunities to find promising all-organic pyroelectrics. In TGS and other organic-inorganic hybrid pyroelectrics, the inorganic parts have negligible contributions to the total dipole polarizations in comparison with the organic parts. If both polar cations and polar anions are properly chosen in an organic salt, they are expected to simultaneously contribute to ferroelectricity and pyroelectricity. In addition, we expected the Curie temperature to be higher than room temperature within a range of several dozens of Kelvin and a continuous phase transition from ferroelectric to paraelectric with increasing temperature. This type of transition allows continuous change in the spontaneous polarization, instead of a sharp jump in the vicinity of the T_C, and enables to maintain a large pyroelectric coefficient over a certain temperature range.

In this study, we initially chose the appropriate polar ions that are conducive to the emergence of ferroelectricity. As the smallest organic carboxylate ion, the formate anion is more likely to undergo an order-disorder transition in a crystal. Meanwhile, spherical-like cations with low rotational energy barriers are promising candidates for inducing structural phase transitions.[19,20] Based on these considerations, a novel all-organic ferroelectric amantadine formate (AF) is found; AF is composed of two polar organic ions (Figure S1A) with T_C = 327 K, which is higher than that of TGS (322 K), but still close to room temperature. Structural analysis shows that both ions contributed to emergent ferroelectricity and pyroelectricity. Our measurements indicate that the ferroelectric-paraelectric phase transition in AF belongs to a continuous or second-order phase transition. As a result, the pyroelectric coefficient of AF is $-170\ \mu C/m^2\cdot K$ at 298 K. The room temperature dielectric constants of AF are only 13.5, 11.7, and 10.7, at 1, 10, and 100 kHz, respectively. The F_vs are 0.705, 0.811, and 0.887 m^2/C at the corresponding frequencies, respectively, higher than those of all known organics, and even TGS. In addition, the strain-electric field measurements reveal that the piezoelectric coefficient of AF is -16 pm/V, providing another example of the rare negative longitudinal piezoelectric effect.[21–25] Our findings provide an all-organic material exhibiting high pyroelectric FOMs. AF has a low density of 1.21 g/cm^3 and a low hardness (0.46 GPa). Moreover, it is non-toxic and inexpensive, both in raw materials and in synthesis. These traits make it a potential material for applications in flexible pyroelectric devices.

RESULTS AND DISCUSSION

AF crystals up to 1 cm were grown by evaporating a mixed ethanol solution of amantadine and formic acid (Figures 1A and S1B). No other concomitant products were present, as confirmed by powder X-ray diffraction (Figure S2). Thermogravimetric analysis (TGA) indicates that AF is stable up to approximately 420 K (Figure S3). Although soluble in water, AF is very stable in air (Figure S4). The elastic module and hardness are measured to be 8.74 GPa and 0.46 GPa (Figure 1A), respectively, which are approximately one-third of the values for TGS (Figure S5) and one to two orders of magnitude smaller than the values for inorganic pyroelectrics.[26] The crystal structures are determined from single-crystal X-ray diffraction (SCXRD) data (Tables S1–S3). AF crystallizes in a

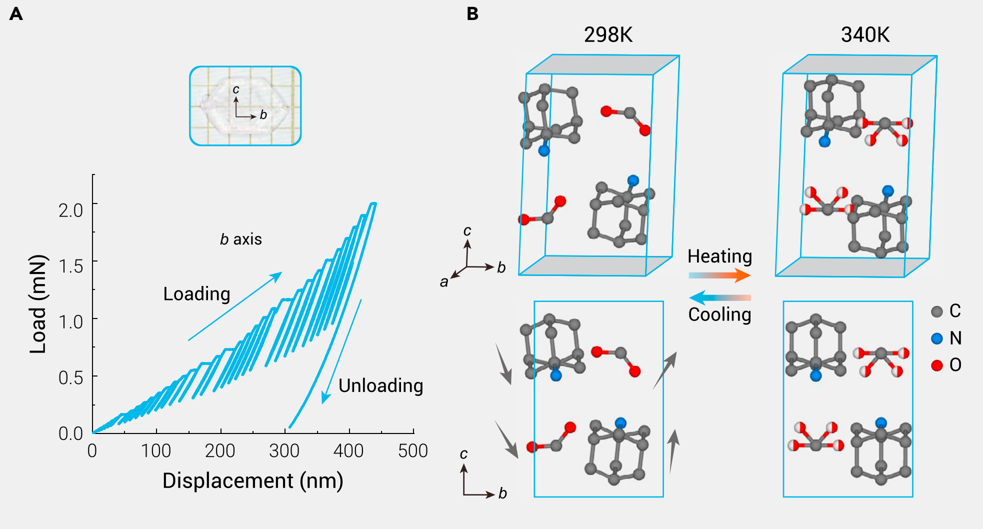

Figure 1. Mechanical properties and crystal structure of AF (A) Optical photograph of a grown single crystal of AF, and the load-displacement curve of AF single crystal along the *b* axis. The fitted elastic module and hardness are 8.74 GPa and 0.46 GPa, respectively. (B) Crystal structures of amantadine formate at 298 K (left) and 340 K (right), where the phase transition temperature is around 327 K. The arrows represent the directions of polarization of ions. The half red and half white balls represent the oxygen atoms with 50% occupation. Hydrogen atoms are omitted for clarity.

monoclinic system with space group $P2_1$ at 298 K with cell parameters a = 8.2200(8) Å, b = 6.5851(7) Å, c = 10.4675(10) Å, β = 106.952(3)°, and V = 541.98(9) Å^3. The calculated density is 1.21 g/cm^3, slightly higher than that of water. The crystal structure is shown in Figure 1B. In the structure, a hydrogen atom of the formic acid molecule is ionized and acquired by the nitrogen atom of amantadine, forming two types of ions in the crystal: $[C_{10}H_{18}N]^+$ and $[HCOO]^-$. Each unit cell contains two AF molecules. There are several N–H···O hydrogen bonds with bond lengths from 2.771 Å to 2.795 Å connecting cations and anions. Both types of ions are polar, and the directions of the dipole moments are shown in Figure 1B. Thus, the net electric dipole moments of both $[C_{10}H_{18}N]^+$ and $[HCOO]^-$ arise along the b axis. The spontaneous polarization of AF can be expressed as $\Sigma p_i/V$ and is along the b axis, where p_i is the electric dipole moment of the ions and V is the volume.

We find that AF undergoes a structural phase transition with increasing temperature. At about 340 K, it recrystallizes in a centrosymmetric structure with space group $P2_1/m$, followed by the disappearance of the spontaneous polarization. The cell becomes slightly larger: a = 8.2566(10) Å, b = 6.6287(6) Å, c = 10.4895(11) Å, β = 107.054(13)°, and V = 548.85(11) Å^3. All $[C_{10}H_{18}N]^+$ cations rotate a little and still remain ordered. Their net dipoles are lost as mirror planes that bisect the molecules now appear, as shown in Figure 1B. The $[HCOO]^-$ anions also rotate and become disordered since they locate at two equivalent orientations connected by a mirror plane perpendicular to the b axis. Similarly, the net moment of the $[HCOO]^-$ anions is also lost. The hydrogen bonds form from the H atoms of the amino groups and random O atoms of the formate anions. Hence, the bond lengths span a slightly larger range, from 2.725 Å to 2.828 Å. This phase transition between point group 2/m and 2 is one of the 88 species of ferroelectrics summarized by Aizu.[27] A displacement of $[C_{10}H_{18}N]^+$ ions and an order-disorder type change of $[HCOO]^-$ ions occur when transiting from the ferroelectric to the paraelectric phase. Therefore, both contribute to polarization below the Curie temperature.

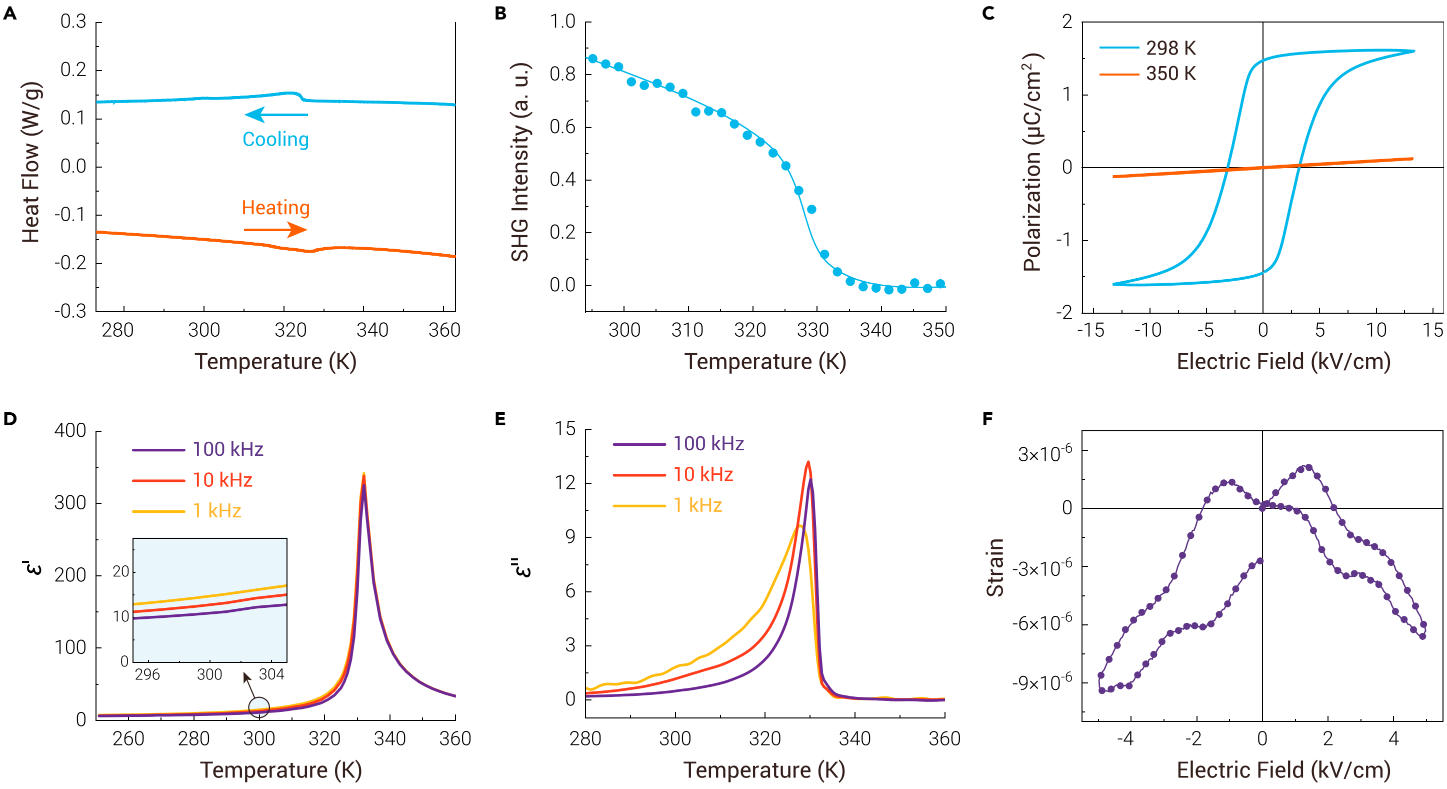

Figure 2. Ferroelectric-related properties (A) DSC data of AF during a heating and cooling cycle, revealing a phase transition around 327 K. (B) Temperature-dependent SHG intensity of polycrystalline sample of AF. (C) Polarization-electric field hysteresis loops along the *b* axis at 298 K and 350 K. The external electric field is a triangle wave with a frequency of 0.01 Hz. (D) Temperature-dependent real part of dielectric constants along the *b* axis at different frequencies. (E) Temperature-dependent imaginary part of dielectric constants along the *b* axis at different frequencies. (F) Strain-electric field hysteresis loop along the *b* axis at 298 K. The external electric field is a triangle wave with a frequency of 100 Hz.

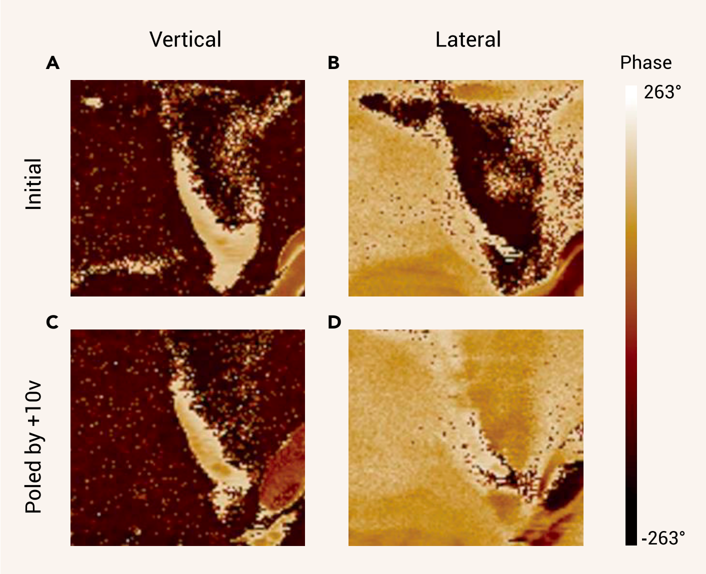

Figure 3. The PFM phase imaging before and after +10 V poling on a thin film of AF (A) The vertical phase before poling.(B) The lateral phase before poling.(C) The vertical phase after poling.(D) The lateral phase after poling. The area for observation and poling is 1 × 1 μm^2. The measured temperature is room temperature.

To confirm this ferroelectric-paraelectric phase transition, we first detected the thermal response of AF. Differential scanning calorimetry (DSC) revealed that a peak appears at 327 K during the heating process, and at 322 K during the cooling process at a temperature change rate of 5 K/min (Figure 2A). Strikingly, in contrast to the λ-shaped peaks (Figure S6) observed in the phase transition for most ferroelectrics,[10,28,29] the peaks of AF are step-like in shape, similar to ferroelectrics TGS,[7] $[C_8H_9N_2]ClO_4$,[30] and $[(CH_3)_3NC_2H_4NH_3]Pb_2Cl_6$.[31] This means that there is only a definite jump in specific heat rather than an indefinite divergence anomaly. Therefore, the phase transition in AF is second-order in nature. Variable-temperature second-harmonic generation (SHG) measurements were also performed to verify the structural phase transition (Figure 2B). Second-harmonic signals are only observed below 330 K, confirming that a phase transition from centrosymmetric to non-centrosymmetric occurs, consistent with the structure determined by SCXRD.

Temperature-dependent dielectric constant was measured on a single crystal. The real part of the dielectric constant (ϵ') along the *b* axis shows λ-shaped anomalies around T_C (Figure 2D), similar to the imaginary part (ϵ'', Figure 2E). However, ϵ' is nearly temperature-independent along the *a* and *c* axes (Figure S7). The Curie-Weiss law is used to depict the behavior of ϵ'. It can be divided into two parts: $\epsilon' = C_{para}/(T - T_C)$ for $T > T_C$ and $\epsilon' = C_{ferro}/(T_C - T)$ for $T < T_C$, where C_{para} and C_{ferro} are Curie-Weiss constants in the paraelectric and ferroelectric phases, respectively. As expected, the reciprocal value of ϵ' for AF as a function of temperature is linear in both the paraelectric and ferroelectric phases, obeying the Curie-Weiss law (Figure S8). The ratio of C_{para}/C_{ferro} for 1 kHz is 2.21, close to the theoretical value of 2 in second-order phase transition ferroelectric.[32] This is again in good agreement with the DSC measurements. At 298 K, ϵ' exhibited a slight frequency dependence (Figure S9A); the values at 1, 10, and 100 kHz are 13.5, 11.7, and 10.7, respectively, always smaller than those of TGS,[2] whereas ϵ'' is comparable to that of TGS, indicating the good insulation of AF (Figure S9B).

One of the most important characteristics of ferroelectrics is the existence of polarization-electric (P-E) hysteresis loops. AF shows a standard P-E hysteresis loop for ferroelectricity at 298 K (Figure 2C). The remnant polarization is approximately 1.47 μC/cm^2, which is larger than 0.25 μC/cm^2 for typical molecular ferroelectric Rochelle salt,[13] but smaller than 3.8 μC/cm^2 for TGS.[33] It should be noted that the polarization slowly decreases after removing the external electric field, indicating the existence of unstable domains. This may contribute to the dielectric dispersion at frequencies lower than 100 kHz.[34,35] The coercive field is about 3.1 kV/cm, smaller than that of most molecular ferroelectrics.[13,36-38] As poling is a necessary procedure for ferroelectrics before being used in pyroelectric devices, such a low coercive field will reduce the cost in the poling process. Meanwhile, the strain-electric field curve exhibits a typical butterfly shape of ferroelectrics and reveals that AF is a negative piezoelectric (Figures 2F and S10), which has attracted much interest recently.[21-25,39,40] The estimated d_{33} is −16 pm/V, consistent with that measured by the Berlincourt method (Figure S11). The value is lower than −37.7 pm/V reported for negative-piezoelectric PVDF[22] and −95 pm/V for $CuInP_2S_6$,[23] but comparable to 6–16 pm/V for positive-piezoelectric $LiNbO_3$,[41] 14 pm/V for $[C_7H_{16}N_2]NH_4I_3$,[42] and 20 pm/V for $[(CH_3)_3NOH]_2KFe(CN)_6$.[43] Furthermore, the structure and switching of domains are observed by piezoresponse force microscopy (PFM), strongly confirming the ferroelectricity of AF (Figure 3).

The temperature dependence of the pyroelectric coefficient was obtained by measuring the current response under temperature ramping from 250 to 360 K. The pyroelectric coefficient first increases and reaches the maximum at T_C. Beyond T_C, it drops rapidly to zero, indicating the occurrence of a polar-nonpolar phase transition (Figure 4A). The polarizations at different temperatures were obtained by integrating the pyroelectric current density over time. At 298 K, the value is 1.44 μC/cm^2, which is in good agreement with that obtained from the P-E hysteresis loop measurement. At temperatures far below the phase transition, the polarization decreases slowly with increasing temperature. When approaching the temperature of the phase transition, the rate of decrease gradually

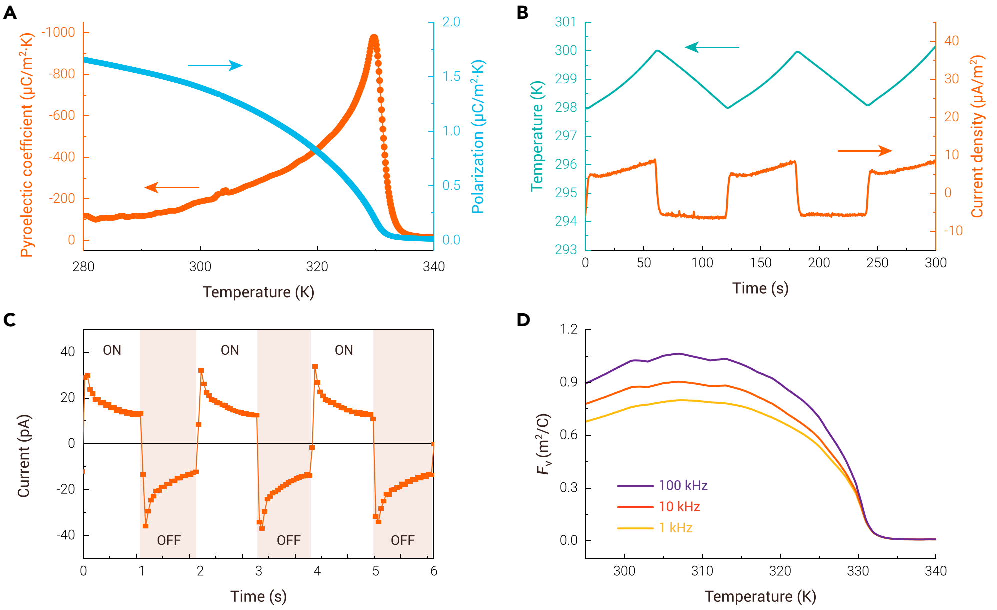

Figure 4. Pyroelectricity of AF (A) Temperature-dependent pyroelectric coefficient and polarization of AF. The latter is obtained by integrating the measured pyroelectric current over time. (B) Current responses under periodic temperature oscillations. (C) Current responses of AF under a periodically switched incandescent lamp. All the current measurements are along the *b* axis. (D) Temperature dependence of pyroelectric voltage FOM (F_v).

accelerates. As a result, large pyroelectric coefficients can exist in this temperature range (−118 μC/m^2·K at 290 K to about −440 μC/m^2·K at 320 K). We also measured the current response under periodic temperature oscillations around 298 K,[44,45] and obtained a pyroelectric coefficient of approximately −170 μC/m^2·K, which is close to the value obtained by temperature ramping. The corresponding pyroelectric current FOM is 0.83 × 10^{-10} m/V. Here, the volume specific heat measured by DSC is adopted in the calculation

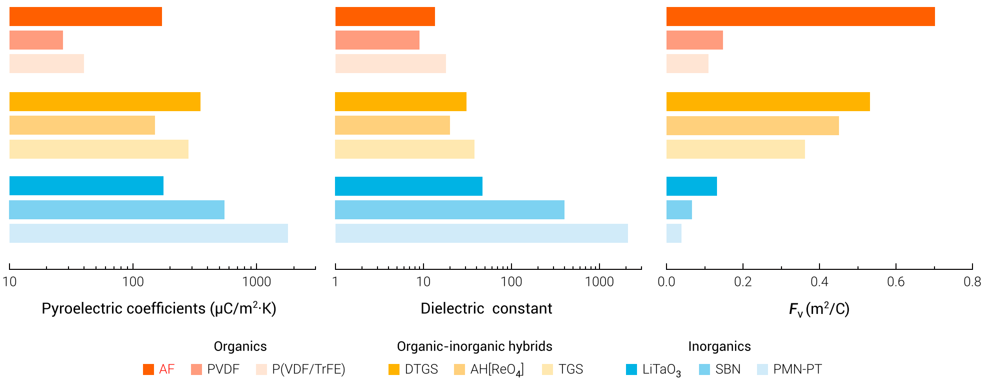

Figure 5. Comparison of pyroelectric coefficients, dielectric constants, and F_vs between AF in this work and some famous pyroelectrics Where PVDF, P(VDF-TrFE), DTGS, SBN, and PMN-PT represent polyvinylidene fluoride, poly(50% vinylidene fluoride-50% trifluoroethylene), deuterated triglycine sulfate, $Sr_{0.5}Ba_{0.5}Nb_2O_6$, and $0.75Pb(Mg_{1/3}Nb_{2/3})O_3$-$0.25PbTiO_3$, respectively.[2,48]

(Table S4). The room temperature pyroelectric coefficient of AF is comparable to that of perovskite oxides with polarization one order of magnitude larger than that of AF: $LiTaO_3$ (−176 μC/m²·K),[1] $PbTiO_3$ (−180 μC/m²·K),[46] and $BaTiO_3$ (−200 μC/m²·K).[47] This is due to the second-order phase transition and suitable T_C. In ferroelectrics, both first- and second-order ferroelectric-paraelectric phase transitions provide large pyroelectric coefficients near T_C. However, the polarization-temperature relationships of these two phase-transition types are different, resulting in different values of the pyroelectric coefficient. According to Landau theory,[32] the order parameter P_r can be described by the following relations for the two types of phase transitions:

$$P_r = \left[\frac{-\beta + \sqrt{\beta^2 - 4\alpha_0\gamma(T - T_c)}}{2\gamma}\right]^{1/2} \qquad \text{(Equation 1)}$$

and

$$P_r = \left[\frac{\alpha_0(T_c - T)}{\beta}\right]^{1/2} \qquad \text{(Equation 2)}$$

where α_0, β, and γ are constants. As shown in Figure S12, at temperatures $T < T_C$, the second-order phase transition ferroelectric has a larger pyroelectric coefficient than the first-order one if they have the same remnant polarization. The temperature dependence of the polarization of AF agrees well with the $\sim[T_C - T]^{1/2}$ law near T_C (Figure S13), confirming the second-order phase transition again. As an illustration, electrical currents are easily induced when irradiating an AF crystal by an incandescent lamp and vary synchronously with alternating switching (Figure 4C), confirming the sensibility to small changes in temperature.

Although those perovskite oxide ferroelectrics were found to have large pyroelectric coefficients, their F_v values are low (usually <0.1 m²/C) because of their large dielectric constants,[2] whereas in AF, small dielectric constant and moderate volume specific heat (Figure S14) resulted in high values of F_v (Figure 4D). At 298 K, F_vs are 0.705, 801, and 0.887 m²/C at 1, 10, and 100 kHz, respectively. These F_v values are considerably higher than those of most inorganic perovskites and even higher than that of TGS (Figure 5). In addition, other dielectric-constant-related pyroelectric FOMs of AF, such as detection capacity FOM (2.17×10^{-5} $Pa^{-1/2}$) and energy harvesting FOM (5.94×10^{-11} m³/J), are comparable to those of commercial pyroelectric materials (Figure S15, Table S4).[49] Thus, AF has a great potential to be used in highly sensitive thermal sensors.[2] It should be noted that the secondary pyroelectric coefficients can be affected by piezoelectric behaviors through the expression $d_{ijk}c_{jklm}\alpha_{lm}$, where d, c, and α are piezoelectric, elastic, and thermal expansion coefficients, respectively.[50] Here, a negative value of d_{33} and a positive value of α_{33} result in a negative component of secondary pyroelectric coefficient −2.8 μC/m²·K, where c = 8.74 GPa (Figure 1B) and $\alpha = 2 \times 10^{-5}$ K^{-1} (Figure S16) are used for the calculation. The total pyroelectric coefficient is slightly enhanced because the primary and secondary coefficients have the same signs. But on the other side, this may cause noise in some applications. In the present case, the noise amplitude is about 1.6%, which is negligibly small. Moreover, the polarization of the negative piezoelectric was found to be abnormally enhanced under stress ($\Delta P = d_{33}\sigma_{33}$, where σ_{33} is the stress).[39] For AF, its polarization will nearly double under a stress of 1 GPa. The pyroelectric coefficient of AF is expected to be further enhanced under pressure.

CONCLUSIONS

In summary, we discovered an all-organic ferroelectric, AF, which shows a high pyroelectric FOM around room temperature, where the F_v is higher than that of all known organic pyroelectrics, even TGS. A higher pyroelectric voltage output is expected in AF. The key to the high F_v is attributed to the continuous phase transition from ferroelectric to paraelectric and low dielectric constant. Meanwhile, AF is a new example for negative piezoelectric effect. Like TGS, the pyroelectric performance of AF can be further improved through molecular doping or molecular modification. Owing to the low density, low hardness, and low cost of this all-organic material, AF is expected to find a great potential to be applied in flexible pyroelectric devices.

MATERIALS AND METHODS

The details of sample preparation and characterization are described in the supplemental information.

REFERENCES

1. Whatmore, R.W. (1986). Pyroelectric devices and materials. Rep. Prog. Phys. ***49***, 1335–1386.
2. Bowen, C.R., Taylor, J., LeBoulbar, E., et al. (2014). Pyroelectric materials and devices for energy harvesting applications. Energy Environ. Sci. ***7***, 3836–3856.
3. Muralt, P. (2001). Micromachined infrared detectors based on pyroelectric thin films. Rep. Prog. Phys. ***64***, 1339–1388.
4. Fuflyigin, V., Salley, E., Osinsky, A., and Norris, P. (2000). Pyroelectric properties of AlN. Appl. Phys. Lett. ***77***, 3075–3077.
5. Yang, M.M., Luo, Z.D., Mi, Z., et al. (2020). Piezoelectric and pyroelectric effects induced by interface polar symmetry. Nature ***584***, 377–381.
6. Matthias, B.T., Miller, C.E., and Remeika, J.P. (1956). Ferroelectricity of glycine sulfate. Phys. Rev. ***104***, 849–850.
7. Hoshino, S., Mitsui, T., Jona, F., and Pepinsky, R. (1957). Dielectric and thermal study of tri-glycine sulfate and tri-glycine fluoberyllate. Phys. Rev. ***107***, 1255–1258.
8. Harada, J., Kawamura, Y., Takahashi, Y., et al. (2019). Plastic/ferroelectric crystals with easily switchable polarization: low-voltage operation, unprecedentedly high pyroelectric performance, and large piezoelectric effect in polycrystalline forms. J. Am. Chem. Soc. ***141***, 9349–9357.
9. Horiuchi, S., Tokunaga, Y., Giovannetti, G., et al. (2010). Above-room-temperature ferroelectricity in a single-component molecular crystal. Nature ***463***, 789–792.
10. Fu, D.W., Cai, H.L., Liu, Y.M., et al. (2013). Diisopropylammonium bromide is a high-temperature molecular ferroelectric crystal. Science ***339***, 425–428.
11. You, Y.M., Liao, W.Q., Zhao, D.W., et al. (2017). An organic-inorganic perovskite ferroelectric with large piezoelectric response. Science ***357***, 306–309.
12. Liao, W.Q., Zhao, D.W., Tang, Y.Y., et al. (2019). A molecular perovskite solid solution with piezoelectricity stronger than lead zirconate titanate. Science ***363***, 1206–1210.
13. Horiuchi, S., and Tokura, Y. (2008). Organic ferroelectrics. Nat. Mater. ***7***, 357–366.
14. Park, C., Lee, K., Koo, M., and Park, C. (2020). Soft ferroelectrics enabling high-performance intelligent photo electronics. Adv. Mater. ***33***, e2004999.
15. Li, Y., Yu, L., Chen, L., et al. (2021). Subtle side chain triggers unexpected two-channel charge transport property enabling 80% fill factors and efficient thick-film organic photovoltaics. The Innovation ***2***, 100090. https://doi.org/10.1016/j.xinn.2021.100090.
16. Liu, X., Pang, H., Liu, X., et al. (2021). Orderly porous covalent organic frameworks-based materials: superior adsorbents for pollutants removal from aqueous solutions. The Innovation ***2***, 100076. https://doi.org/10.1016/j.xinn.2021.100076.
17. Szklarz, P., and Bator, G. (2005). Pyroelectric properties of tricyclohexylmethanol (TCHM) single crystal. J. Phys. Chem. Sol. ***66***, 121–125.
18. Sun, Z., Tang, Y., Zhang, S., et al. (2015). Ultrahigh pyroelectric figures of merit associated with distinct bistable dielectric phase transition in a new molecular compound: di-n-butylaminium trifluoroacetate. Adv. Mater. ***27***, 4795–4801.
19. Zhang, H.Y., Tang, Y.Y., Shi, P.P., and Xiong, R.G. (2019). Toward the targeted design of molecular ferroelectrics: modifying molecular symmetries and homochirality. Acc. Chem. Res. ***52***, 1928–1938.

20. Liu, H.Y., Zhang, H.Y., Chen, X.G., and Xiong, R.G. (2020). Molecular design principles for ferroelectrics: ferroelectrochemistry. J. Am. Chem. Soc. ***142***, 15205–15218.
21. Katsouras, I., Asadi, K., Li, M., et al. (2015). The negative piezoelectric effect of the ferroelectric polymer poly(vinylidene fluoride). Nat. Mater. ***15***, 78–84.
22. You, L., Zhang, Y., Zhou, S., et al. (2019). Origin of giant negative piezoelectricity in a layered van der Waals ferroelectric. Sci. Adv. ***5***, eaav3780.
23. Kim, J., Rabe, K.M., and Vanderbilt, D. (2019). Negative piezoelectric response of van der Waals layered bismuth tellurohalides. Phys. Rev. B ***100***, 104115.
24. Neumayer, S.M., Eliseev, E.A., Susner, M.A., et al. (2019). Giant negative electrostriction and dielectric tunability in a van der Waals layered ferroelectric. Phys. Rev. Mater. ***3***, 024401.
25. Liu, Y., and Wang, Q. (2020). Ferroelectric polymers exhibiting negative longitudinal piezoelectric coefficient: progress and prospects. Adv. Sci. ***7***, 1902468.
26. Xie, Y., Ai, Y., Zeng, Y.L., et al. (2020). The soft molecular polycrystalline ferroelectric realized by the fluorination effect. J. Am. Chem. Soc. ***142***, 12486–12492.
27. Aizu, K. (1966). Possible species of ferroelectrics. Phys. Rev. ***146***, 423–429.
28. Liao, W.Q., Zhang, Y., Hu, C.L., et al. (2015). A lead-halide perovskite molecular ferroelectric semiconductor. Nat. Commun. ***6*** (7), 7338.
29. Pan, Q., Liu, Z.B., Tang, Y.Y., et al. (2017). A three-dimensional molecular perovskite ferroelectric: (3-Ammoniopyrrolidinium)$RbBr_3$. J. Am. Chem. Soc. ***139***, 3954–3957.
30. Cai, H.L., Zhang, W., Ge, J.Z., et al. (2011). 4-(cyanomethyl)anilinium perchlorate: a new displacive-type molecular ferroelectric. Phys. Rev. Lett. ***107***, 147601.
31. Zhang, H.Y., Song, X.J., Cheng, H., et al. (2020). A three-dimensional lead halide perovskite-related ferroelectric. J. Am. Chem. Soc. ***142***, 4604–4608.
32. Devonshire, A.F. (1954). Theory of ferroelectrics. Adv. Phys. ***3***, 85–130.
33. Lal, R.B., and Batra, A.K. (1993). Growth and properties of triglycine sulfate (TGS) crystals: review. Ferroelectrics ***142***, 51–82.
34. Kikuta, T., Yamada, T., Yamazaki, T., and Nakatani, N. (2010). Dielectric dispersion of triglycine sulfate at ferroelectric phase. Ferroelectrics ***272***, 351–356.
35. Jones, J.L., Aksel, E., Tutuncu, G., et al. (2012). Domain wall and interphase boundary motion in a two-phase morphotropic phase boundary ferroelectric: frequency dispersion and contribution to piezoelectric and dielectric properties. Phys. Rev. B ***86***, 024104.
36. Hang, T., Zhang, W., Ye, H.Y., and Xiong, R.G. (2011). Metal-organic complex ferroelectrics. Chem. Soc. Rev. ***40***, 3577–3598.
37. Zhang, W., and Xiong, R.G. (2012). Ferroelectric metal-organic frameworks. Chem. Rev. ***112***, 1163–1195.
38. Shi, P.P., Tang, Y.Y., Li, P.F., et al. (2016). Symmetry breaking in molecular ferroelectrics. Chem. Soc. Rev. ***45***, 3811–3827.
39. Liu, S., and Cohen, R.E. (2017). Origin of negative longitudinal piezoelectric effect. Phys. Rev. Lett. ***119***, 207601.
40. Qi, Y., and Rappe, A.M. (2021). Widespread negative longitudinal piezoelectric responses in ferroelectric crystals with layered structures. Phys. Rev. Lett. ***126***, 217601.
41. Weis, R.S., and Gaylord, T.K. (1985). Lithium niobate: summary of physical properties and crystal structure. Appl. Phys. A. ***37***, 191–203.
42. Ye, H.Y., Tang, Y.Y., Li, P.F., et al. (2018). Metal-free three-dimensional perovskite ferroelectrics. Science ***361***, 151–155.
43. Xu, W.J., Li, P.F., Tang, Y.Y., et al. (2017). A molecular perovskite with switchable coordination bonds for high-temperature multiaxial ferroelectrics. J. Am. Chem. Soc. ***139***, 6369–6375.
44. Lubomirsky, I., and Stafsudd, O. (2012). Invited review article: practical guide for pyroelectric measurements. Rev. Sci. Instrum. ***83***, 051101.
45. Jachalke, S., Mehner, E., Stöcker, H., et al. (2017). How to measure the pyroelectric coefficient? Appl. Phys. Rev. ***4***, 021303.
46. Takenaka, T., and Sakata, K. (1989). Piezoelectric and pyroelectric properties of calcium-modified and grain-oriented $(NaBi)_{1/2}Bi_4Ti_4O_{15}$ ceramics. Ferroelectrics ***94***, 175–181.
47. Bowen, C.R., Kim, H.A., Weaver, P.M., and Dunn, S. (2014). Piezoelectric and ferroelectric materials and structures for energy harvesting applications. Energy Environ. Sci. ***7***, 25–44.
48. Felix, P., Gamot, P., Lacheau, P., and Raverdy, Y. (1977). Pyroelectric, dielectric and thermal properties of TGS, DTGS and TGFB. Ferroelectrics ***17***, 543–551.
49. Bowen, C.R., Taylor, J., Le Boulbar, E., et al. (2015). A modified figure of merit for pyroelectric energy harvesting. Mater. Lett. ***138***, 243–246.
50. Bhalla, A.S., and Newnham, R.E. (1980). Primary and secondary pyroelectricity. Phys. Status Solidi A ***58***, K19–K24.

ACKNOWLEDGMENTS

We thank Y. T. Song, C. C. Zhao, Y. T. Wang, and C. M. Li for their assistances in the single-crystal X-ray diffraction, dielectric measurements, nano-indentation measurements, and PFM measurements, respectively. This work is financially supported by the National Key Research and Development Program of China (2018YFE0202600, 2016YFA0300301), the National Natural Science Foundation of China under granting numbers: No. 51532010, 91422303, 51772323; and the Key Research Program of Frontier Sciences, CAS, Grant No. QYZDJ-SSW-SLH013.

AUTHOR CONTRIBUTIONS

X.C. and S.J. conceived the experiment and supervised the project. J.Z. prepared the sample and measured the properties with help from C.C., M.H., and X.Z. J.Z. wrote the primary manuscript with input from all authors. X.C., T.Y., and J.G. revised the manuscript.

DECLARATION OF INTERESTS

The authors declare no competing interests.

LEAD CONTACT WEBSITE

http://a02.iphy.ac.cn/

SUPPLEMENTAL INFORMATION

Supplemental information can be found online at https://doi.org/10.1016/j.xinn.2021.100204.

The Innovation, Volume *3*

Supplemental Information

Discovery of amantadine formate: Toward achieving ultrahigh pyroelectric performances in organics

Junyan Zhou, Shifeng Jin, Congcong Chai, Munan Hao, Xin Zhong, Tianping Ying, Jiangang Guo, and Xiaolong Chen

MATERIAL AND METHODS

Sample Preparations

In a typical run for amantadine formate (AF), 6 g of amantadine (Innochem, 98 wt. %) and 3 ml of formic acid (Innochem, 99 wt.%) were mixed into 50 ml anhydrous ethanol to get a clear and transparent solution. Dozens of crystals of AF with varying sizes were obtained by slowly evaporating the solvent at room temperature for about a week. Large, transparent and colorless crystals up to 1 cm were selected for property measurements. The crystals of triglycine sulfate (TGS) were preparation by slow cooling according to the literature.[1] Powder of barium titanate (99.9 wt. %) was purchased from Innochem. Ceramic of modified lead zirconate titanate (PIC 151) was purchased from Physik Instrumente.

The thin film of AF was grown by spin-coating method. The precursor solution is a saturated alcohol solution of AF. Then, 20 μL precursor solution was spread on a clean ITO-coated glass substrate. The thin films were obtained after spin-coating (1500 r/min) for 30 s and annealing at 313 K for 30 min.

Crystal structures determination

Single crystal diffraction data were collected using a Bruker D8 VENTURE PHOTO II diffractometer and a Rigaku XtaLAB Synergy R diffractometer for 298 K and 340 K, respectively. Both of the sources were multilayer mirror monochromatized Mo Kα (λ = 0.71073 Å) radiation. Data collection, cell refinement, and data reduction were carried out in the Bruker APEX program for 298 K and Rigaku CrysAlis PRO program for 340 K. The structures were solved by direct methods and refined by the full-matrix method based on F^2 using the SHELXTL software package.[2] All non-hydrogen atoms were refined anisotropically, and the H atoms were placed in geometrically idealized positions. Experimental details are listed in Table S1. CCDC 2077688 and 2077689 contain the supplementary crystallographic data for this paper. These data can be obtained free of charge from The Cambridge Crystallographic Data Centre via www.ccdc.cam.ac.uk/data_request/cif.

Variable temperature PXRD measurements were performed on a Rigaku SmartLab diffractometer with Cu Kα radiation (λ = 1.5406 Å, 40 kV, 30 mA) and a graphite monochromator in a reflection mode (2θ = 5° to 80°, step = 0.01 °2θ, and scan speed = 1 °·min^{-1}). Indexing and Rietveld refinements were performed using the DICVOL91 and FULLPROF programs, respectively.[3,4]

Thermal properties

Thermogravimetric analyses (TGA) were carried out using TA SDTQ600 thermal analyzer under Ar atmosphere in the temperature range of 20−350 °C in alumina crucibles with a heating rate of 10

K·min^{-1}. Differential scanning calorimetry (DSC) measurements were carried out using a TA DSCQ200 thermal analyzer under Ar atmosphere with heating and cooling rates of 5 K·min^{-1}.

Elastic modules and hardnesses

Nano-indentation measurements were carried out using Bruker TI 980 triboIndenter. TGS was used to compare with AF, the samples are both single crystals along the *b* axis. The measured elastic modules of TGS and AF are 24.18 GPa and 8.74 GPa, respectively. The fitted hardnesses of TGS and AF are 1.30 GPa and 0.46 GPa, respectively.

Second harmonic generations

The second harmonic generations (SHG) measurements were employed by EKSPLA PL2210A laser beam with low divergence (pulsed Nd:YAG, λ = 1064 nm). The powder of AF was put into a UV cuvette. The reflected light of the sample is received by a spectrometer, and the SHG intensity was obtained from the reflected light intensity around 532 nm. The temperature was controlled by HFS600E LinKam stage.

Dielectric constants

The dielectric constant measurements were employed by Keysight impedance analyzer with an applied ac electric field of 500 mV. Crystals of AF and TGS were cut into a plate perpendicular to the *b* axis, the sizes are 2.0 mm^2 × 0.32 mm for AF and 3.1 mm^2 × 0.32 mm for TGS. Silver paste was coated as electrodes on both sides. The temperature was also controlled by HFS600E LinKam stage.

P-E hysteresis loops

The polarization-electric field (P-E) measurements were performed on aixACCT TF Analyzer 3000 Measurement System. The sample was the same one for dielectric constant measurements. The temperature was controlled by Delta 9023 oven.

Piezoelectricity

We used two methods to measure the piezoelectric coefficients: interferometer and Berlincourt methods. The interferometer method is performed on aixACCT TF Analyzer 3000 Measurement System. The piezoelectric coefficient d_{33} was estimated by measuring the strain-electric field loop. The Berlincourt method is carried out using a ZJ-3AN quasi-static d_{33} meter, the gear position is "×0.1".

Piezoresponse force microscopy measurements

The PFM measurement was carried out on Bruker Nano Inc. atomic force microscope at room temperature. The frequencies of AC drive voltage for both out-plane and in-plane phase imaging were 350 kHz.

Pyroelectricity

The sample for pyroelectric measurements was same for dielectric constant measurements. Before the measurements, it was poled by an electric field of 10 $kV\cdot cm^{-1}$ (coercive field is about 3.1 $kV\cdot cm^{-1}$) for 100 s. The pyroelectric coefficients from 250~350 K were measured by temperature ramping techniques [5]. The sample was firstly cooled to 200 K, After that, it was heated to 360 K with a rate of 2 $K\cdot min^{-1}$. The pyroelectric coefficient equals the pyroelectric current density divided by the heating rate. In addition, periodic temperature change techniques [5] was performed at 298 K. The sample was in a temperature oscillation in the form of triangular wave with amplitude of 2 K and period of 120 s. The current was recorded by aixACCT TF Analyzer 3000 Measurement System, and the temperature was controlled by HFS600E LinKam stage.

The sample for measuring the electric response to surface illumination was same for pyroelectric measurements. A incandescent lamp was used to generating periodically illumination. The illumination area was much larger than the sample size.

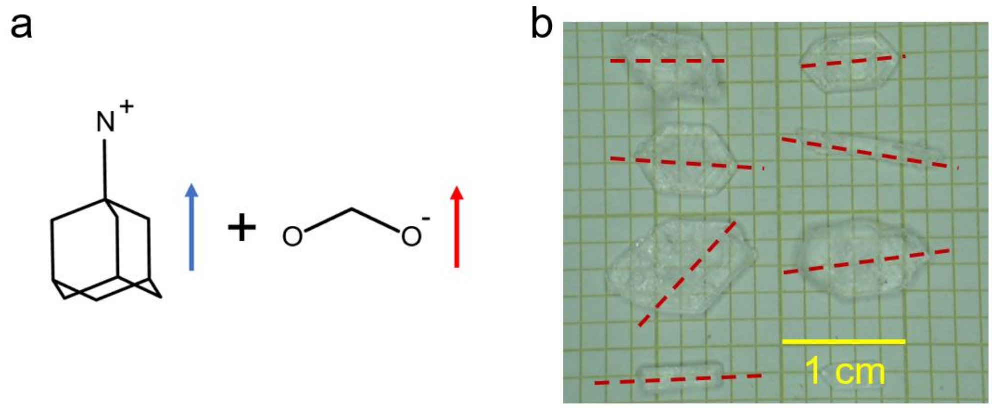

Fig. S1 a, Schematic structures of amantadine (left) and formate (right) ions in AF. The bond types of C-O are not distinguished in formate. Both ions are polar and the directions of their dipole moments are represented by blue and red arrows. **b,** Optical photograph of the grown single crystals of AF. The red dashed lines represent the *b* axis of the crystals, and the *c* axis is perpendicular to the grid paper.

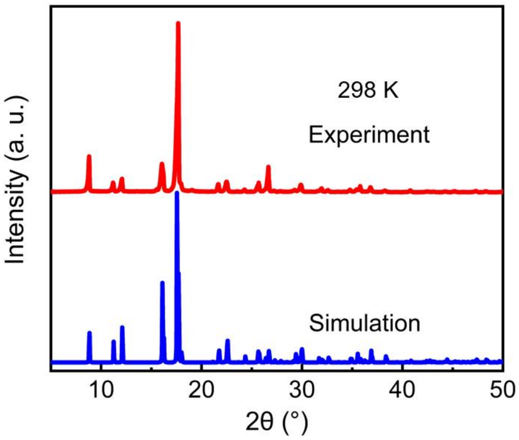

Fig. S2 Powder X-ray diffraction pattern of amantadine formate at 298 K, the blue curve is simulated from the structure obtained by single crystal X-ray diffraction.

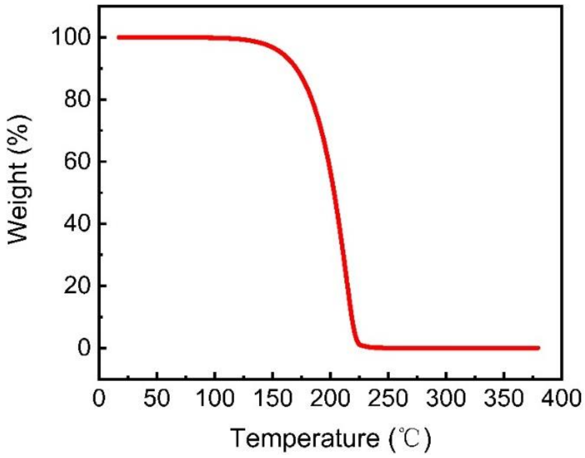

Fig. S3 Thermogravimetry of AF, the decomposition (or sublimation) temperature is about 420 K.

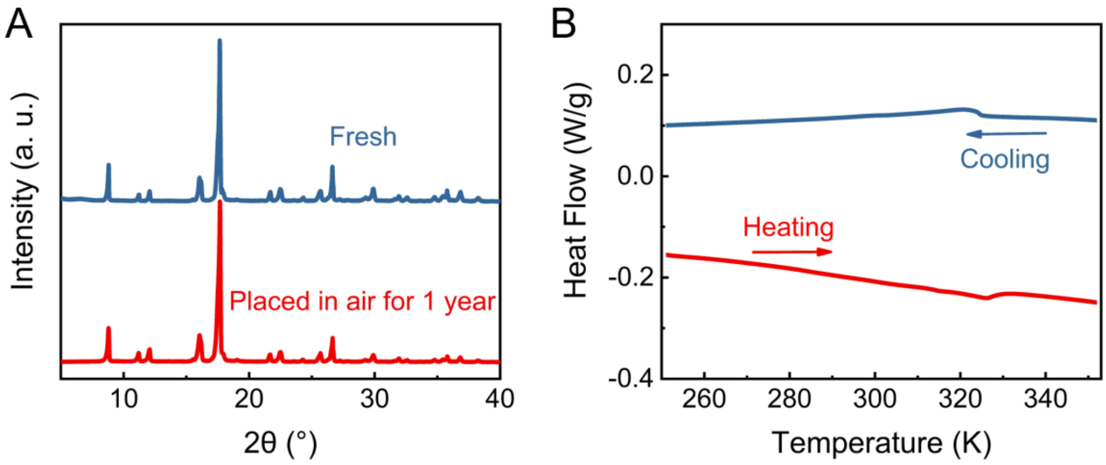

Fig. S4 (A) Comparation of PXRD patterns between AF powder just synthesized and that placed for about one year in air. (B) DSC curves of AF placed for about one year in air. The sample was firstly cooled to 240 K. The heat flow was measured during a 240 K-360 K-240 K cycle in a temperature changing rate of 5 K/min. There is no themal signal of water melting or freezing.

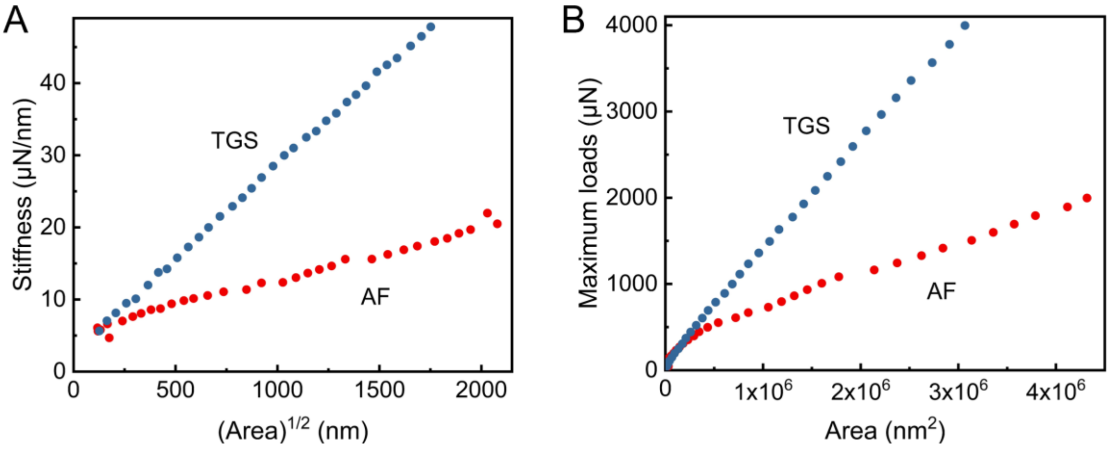

Fig. S5 Results of nano-indentation measurements on single crystals of both TGS and AF. (A) Stiffnesses as functions of the square root of indentation area. The fitted elastic modules of TGS and AF are 24.18 GPa and 8.74 GPa, respectively. (B) Maximum loads as functions of indentation area. The fitted hardnesses of TGS and AF are 1.30 GPa and 0.46 GPa, respectively. Both are measured along the *b* axis.

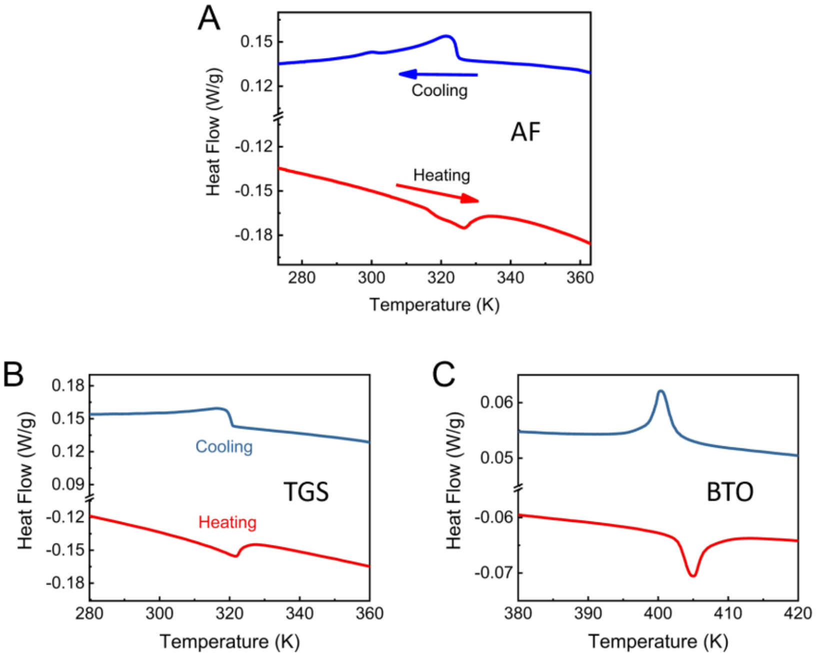

Fig. S6 DSC curves of ferroelectrics. (A) AF in this work. (B) TGS. (C) $BaTiO_3$. Where $BaTiO_3$ and TGS are well-known ferroelectrics with first-order phase transition and second-order phase transition, respectively.

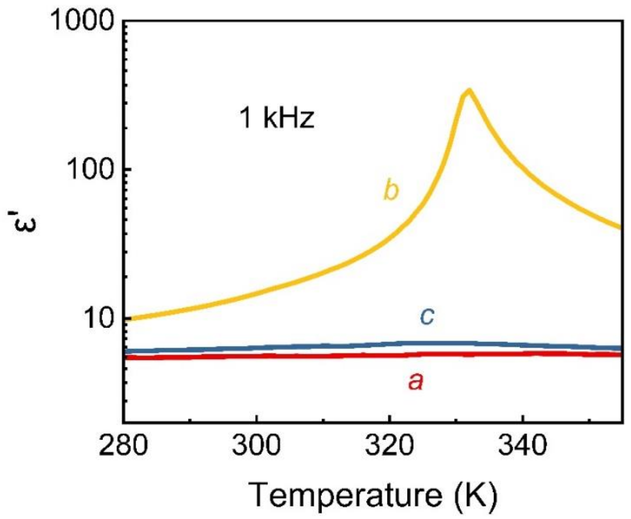

Fig. S7 Anisotropic real part of dielectric constants of AF.

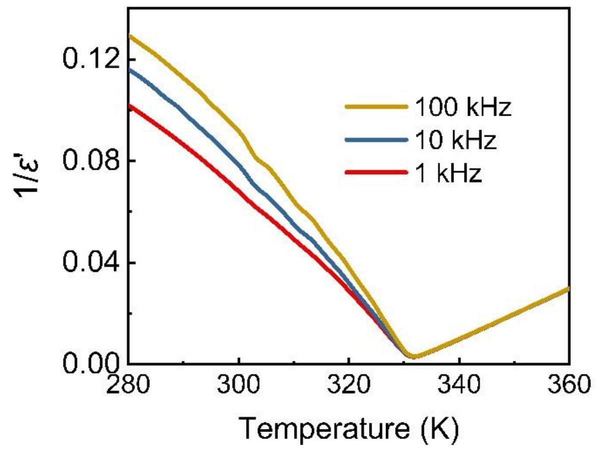

Fig. S8 Temperature dependence of the reciprocal of dielectric constants for AF at several frequencies.

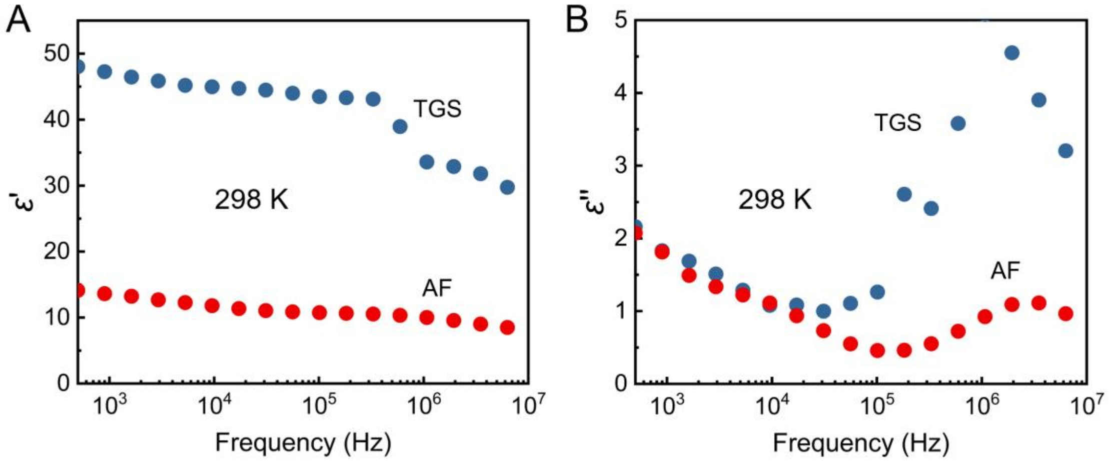

Fig. S9 Dispersion of complex dielectric constants at 298 K for TGS and AF (A) Real part. (B) Imaginary part. Both are measured along the *b* axis.

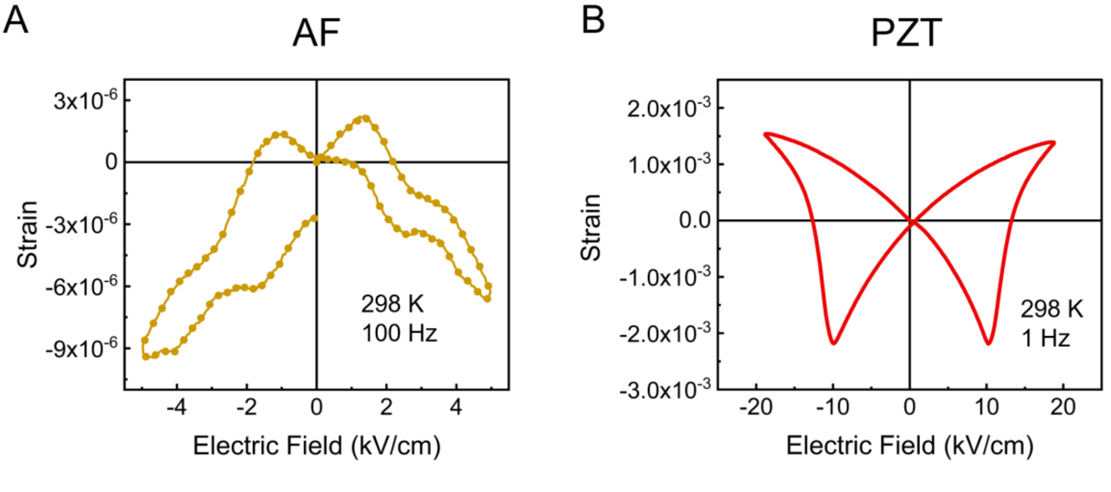

Fig. S10 Strain-electric field curves of (A) AF (*b* axis) in this work and (B) modified PZT. The latter is a positive piezoelectric. The positive value of strain represents elongation, while negative for shortening. The estimated piezoelectric coefficients are −16 pm·V^{-1} for AF and 797 pm·V^{-1} for modified PZT. The measured temperature is 298 K, the frequencies are 100 Hz for AF and 1 Hz for modified PZT.

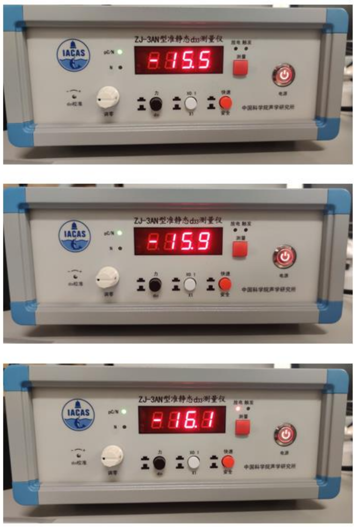

The first time: −15.5 pC/N

The second time: −15.9 pC/N

The third time: −16.1 pC/N

Fig. S11 Results of d_{33} measured by Berlincourt methods, giving a d_{33} value −15.8 ± 0.5 pC/N.

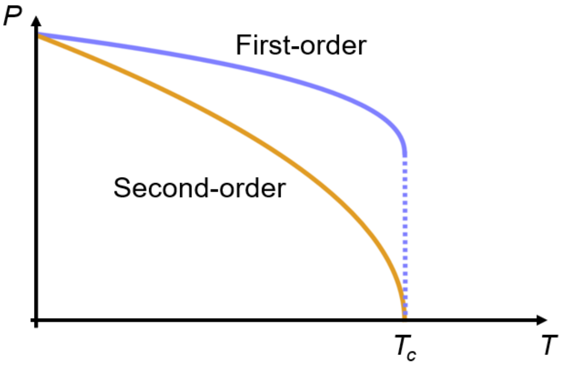

Fig. S12 Comparation of the polarization-temperature behavior of first-order and second-order ferroelectric-paraelectric phase transition based on Landau theory.

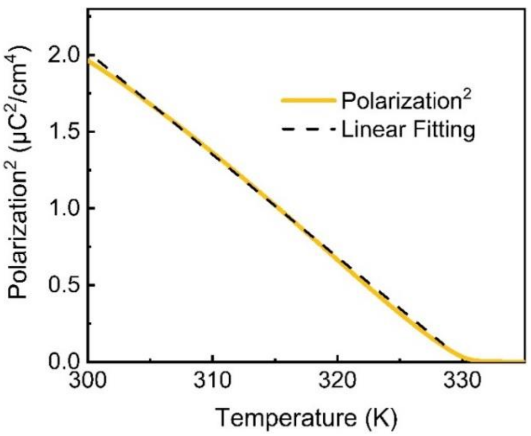

Fig. S13 The relationship between polarization square of AF and temperature. The curve from 300 K to 330 K is well fitted by a straight line, suggests the ~ $[T_C - T]^{1/2}$ law of polarization near T_C.

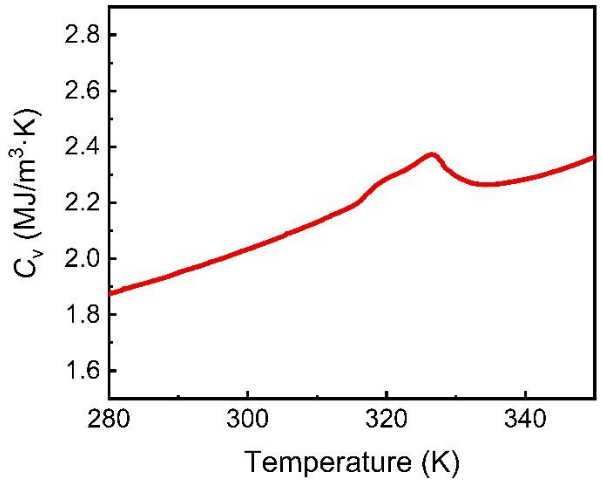

Fig. S14 Temperature-dependent volume specific heat of AF, calculated from the heating run of DSC curve.

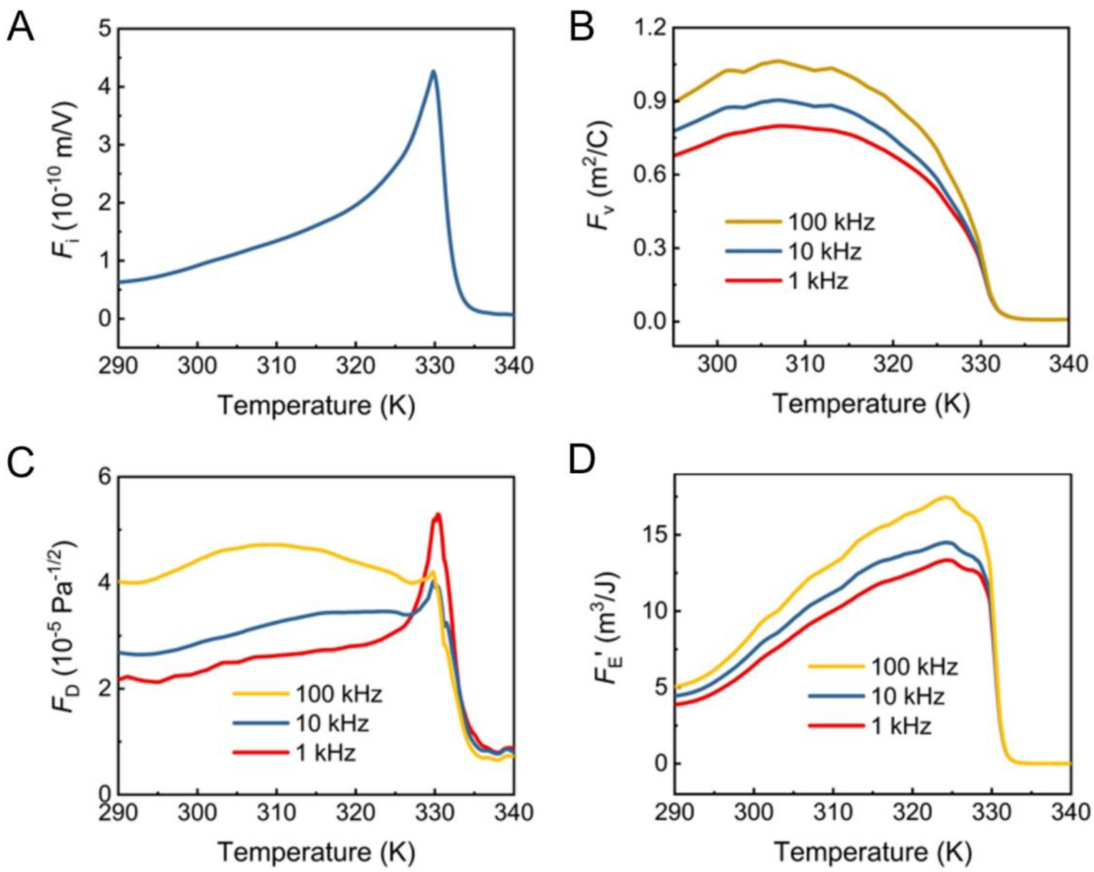

Fig. S15 Temperature-dependent pyroelectric FOMs for current sensitivity (A), voltage responsivity (B), detection capacity (C), and energy harvesting (D).

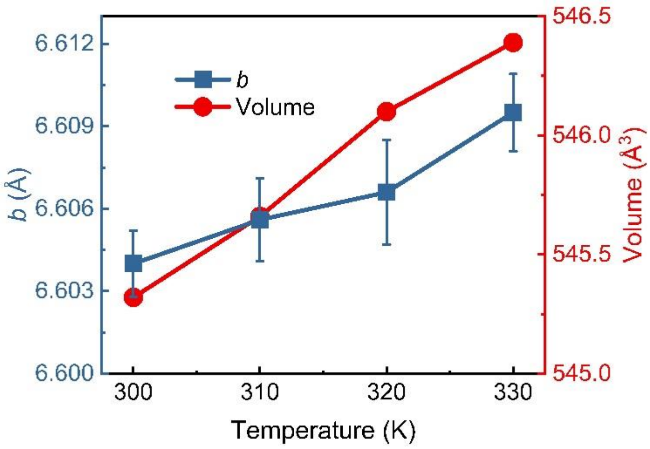

Fig. S16 Temperature dependence of the cell parameter *b* and the unit cell volume of AF. The thermal expansion coefficient along the *b*-axis is estimated to be 2×10^{-5} K^{-1}.

Table S1. Crystal Data of AF at Different Temperatures

Formula	$[C_{10}H_{18}N][HCOO]$	
Formula weight	197.28	
Temperature	298 K	340 K
Crystal system	Monoclinic	
Space group	$P2_1$	$P2_1/m$
a (Å)	8.2200(8)	8.2566(10)
b (Å)	6.5851(7)	6.6287(6)
c (Å)	10.4675(10)	10.4895(11)
β (°)	106.952(3)	107.054(13)
Volume (Å^3)	541.98(9)	548.85(11)
Z	2	2
Density (g·cm^{-3})	1.21	1.19
F(000)	242.0	242.0
θ range for data collection (°)	2.034-28.384	2.580-30.458
Index ranges	$-10 \le h \le 10$<br>$-8 \le k \le 8$<br>$-13 \le l \le 14$	$-10 \le h \le 11$<br>$-9 \le k \le 8$<br>$-13 \le l \le 9$
Independent reflections	2694	1570
Goodness-of-fit on F^2	1.055	0.980
R_1 [$I > 2\sigma(I)$]	0.0574	0.0638
wR_2 [$I > 2\sigma(I)$]	0.1583	0.1876

$R_1 = \sum||F_o| - |F_c||/\sum|F_o|$, $wR_2 = \{\sum[w(|F_o|^2 - |F_c|^2)]/\sum[w|F_o|^4]\}^{1/2}$

Table S2. Fractional atomic coordinates, equivalent isotropic displacement parameters and occupancy rates for non-hydrogen atoms in AF at 298 K.

Atom	*x*	*y*	*z*	U_{iso}	Occupancy rate
C1	0.8696(3)	0.1531(3)	0.6888(2)	0.0392(4)	1
C2	0.9520(3)	0.1290(4)	0.8386(2)	0.0483(6)	1
C3	0.7558(3)	-0.0271(4)	0.6331(3)	0.0489(6)	1
C4	0.7654(3)	0.3494(3)	0.6624(3)	0.0478(6)	1
C5	0.6249(4)	0.3361(4)	0.7308(3)	0.0547(6)	1
C6	0.6162(4)	-0.0402(4)	0.7024(3)	0.0551(7)	1
C7	0.8117(4)	0.1147(4)	0.9072(2)	0.0559(7)	1
C8	0.6983(5)	-0.0662(5)	0.8530(3)	0.0637(8)	1
C9	0.7061(4)	0.3084(5)	0.8811(3)	0.0624(7)	1
C10	0.5110(3)	0.1530(5)	0.6767(3)	0.0615(7)	1
C11	0.2227(3)	0.6670(4)	0.7361(2)	0.0468(5)	1
N1	1.0073(3)	0.1666(3)	0.6212(2)	0.0483(5)	1
O1	0.2190(3)	0.4822(4)	0.7429(3)	0.0810(8)	1
O2	0.1468(4)	0.7766(4)	0.6427(2)	0.0736(7)	1

Table S3. Fractional atomic coordinates, equivalent isotropic displacement parameters and occupancy rates for non-hydrogen atoms in AF at 340 K.

Atom	*x*	*y*	*z*	U_{iso}	Occupancy rate
C1	0.3701(3)	0.2500	0.6888(2)	0.0604(6)	1
C2	0.2616(2)	0.0622(3)	0.64769(18)	0.0759(6)	1
C3	0.1229(2)	0.0635(3)	0.71688(19)	0.0832(6)	1
C4	0.0135(3)	0.2500	0.6760(3)	0.0891(9)	1
C5	0.3133(4)	0.2500	0.9070(2)	0.0884(9)	1
C6	0.2039(3)	0.0639(3)	0.8669(2)	0.0979(8)	1
C7	0.4524(3)	0.2500	0.8386(2)	0.0729(7)	1
C8	0.2773(3)	0.2500	0.2658(2)	0.0731(8)	1
N1	0.5072(3)	0.2500	0.62081(19)	0.0754(7)	1
O1	0.3569(4)	0.1277(5)	0.3568(3)	0.0986(11)	0.5
O2	0.2800(4)	0.0775(5)	0.2651(4)	0.1070(11)	0.5

Table S4. Comparison of room-temperature pyroelectric-related properties between AF and other famous pyroelectrics. Where $F_i = p/C_v$, $F_v = p/\varepsilon' C_v$, $F_D = p/C_v(\varepsilon'')^{1/2}$, $F_E' = p^2/\varepsilon'(C_v)^2$.

	Materials	p μC/m²·K	C_v MJ/m³·K	ε'	ε''	F_i 10^{-10} m/V	F_v m²/C	F_D 10^{-5} Pa$^{-1/2}$	F_E' 10^{-11} m³/J
Organics	**AF This Work**	**170**	**2.02**	**13.5 (1 kHz)**	**1.7 (1 kHz)**	**0.843**	**0.705**	**2.17**	**5.94**
	PVDF[6]	52	2.2	18	0.95	0.24	0.146	0.80	0.35
	P(VDF/TrFE) 90/10[6]	80	2.6	22	0.90	0.31	0.159	1.1	0.49
Organic-inorganic hybrids	DTGS[7]	400	2.4	33	0.66	1.67	0.57	6.9	9.52
	AH[ReO$_4$][8]	150	1.84	20.5	0.5	0.82	0.45	3.84	3.69
	TGS[7]	350	2.6	40	1.0	1.35	0.38	4.5	5.13
Inorganics	LaTiO$_3$[9]	190	3.2	47	0.24	0.59	0.14	4.1	0.83
	SBN[9]	550	2.2	400	1.2	2.48	0.07	7.2	1.74
	PMN-0.13PT[10]	3260	2.6	3107	10.6	13.0	0.046	13.5	5.98

References

1. Senthil Pandian, M., Verma, S., Karuppasamy, P., et al. (2020). TGS crystal growth below and above Curie temperature (Tc). J. Cryst. Growth ***546***, 125793, 10.1016/j.jcrysgro.2020.125793.
2. Sheldrick, G.M. (2008). A short history of SHELX. Acta Crystallogr. A ***64***, 112-122, 10.1107/S0108767307043930.
3. Louër, D., and Louër, M. (1972). Méthode d'essais et erreurs pour l'indexation automatique des diagrammes de poudre. J. Appl. Crystallogr. ***5***, 271-275, 10.1107/s0021889872009483.
4. Rodríguez-Carvajal, J. (1993). Recent advances in magnetic structure determination by neutron powder diffraction. Physica B ***192***, 55-69, 10.1016/0921-4526(93)90108-i.
5. Lubomirsky, I., and Stafsudd, O. (2012). Invited review article: practical guide for pyroelectric measurements. Rev. Sci. Instrum. ***83***, 051101, 10.1063/1.4709621.
6. Dietze, M., Krause, J., Solterbeck, C.H., and Es-Souni, M. (2007). Thick film polymer-ceramic composites for pyroelectric applications. J. Appl. Phys. ***101***, 054113, 10.1063/1.2653978.
7. Felix, P., Gamot, P., Lacheau, P., and Raverdy, Y. (1977). Pyroelectric, dielectric and thermal properties of TGS, DTGS and TGFB. Ferroelectrics ***17***, 543-551, 10.1080/00150197808236779.
8. Harada, J., Kawamura, Y., Takahashi, Y., et al. (2019). Plastic/Ferroelectric Crystals with Easily Switchable Polarization: Low-Voltage Operation, Unprecedentedly High Pyroelectric Performance, and Large Piezoelectric Effect in Polycrystalline Forms. J. Am. Chem. Soc. ***141***, 9349-9357, 10.1021/jacs.9b03369.
9. Whatmore, R.W., Patel, A., Shorrocks, N.M., and Ainger, F.W. (1990). Ferroelectric materials for thermal ir sensors state-of-the-art and perspectives. Ferroelectrics ***104***, 269-283, 10.1080/00150199008223829.
10. Yu, P., Tang, Y., and Luo, H. (2007). Fabrication, property and application of novel pyroelectric single crystals—PMN–PT. J. Electroceram. ***24***, 1-4, 10.1007/s10832-007-9360-7.
